# STRAIGHT-IN enables high-throughput targeting of large DNA payloads in human pluripotent stem cells

**DOI:** 10.1016/j.crmeth.2022.100300

**Published:** 2022-09-22

**Authors:** Albert Blanch-Asensio, Catarina Grandela, Karina O. Brandão, Tessa de Korte, Hailiang Mei, Yavuz Ariyurek, Loukia Yiangou, Mervyn P.H. Mol, Berend J. van Meer, Susan L. Kloet, Christine L. Mummery, Richard P. Davis

**Affiliations:** 1 Department of Anatomy and Embryology, Leiden University Medical Center, 2300RC Leiden, the Netherlands; 2 Department of Applied Stem Cell Technologies, University of Twente, 7500AE Enschede, the Netherlands; 3 Sequencing Analysis Support Core, Leiden University Medical Center, 2333RC Leiden, the Netherlands; 4 Leiden Genome Technology Center, Leiden University Medical Center, 2333RC Leiden, the Netherlands

**Keywords:** site-specific recombination, targeted gene modification, Bxb1 integrase, Cre recombinase, CRISPR-Cas9, human pluripotent stem cells, disease modeling, synthetic gene circuit, cardiomyocyte

## Abstract

Inserting large DNA payloads (>10 kb) into specific genomic sites of mammalian cells remains challenging. Applications ranging from synthetic biology to evaluating the pathogenicity of disease-associated variants for precision medicine initiatives would greatly benefit from tools that facilitate this process. Here, we merge the strengths of different classes of site-specific recombinases and combine these with CRISPR-Cas9-mediated homologous recombination to develop a strategy for stringent site-specific replacement of genomic fragments at least 50 kb in size in human induced pluripotent stem cells (hiPSCs). We demonstrate the versatility of STRAIGHT-IN (serine and tyrosine recombinase-assisted integration of genes for high-throughput investigation) by (1) inserting various combinations of fluorescent reporters into hiPSCs to assess the excitation-contraction coupling cascade in derivative cardiomyocytes and (2) simultaneously targeting multiple variants associated with inherited cardiac arrhythmic disorders into a pool of hiPSCs. STRAIGHT-IN offers a precise approach to generate genetically matched panels of hiPSC lines efficiently and cost effectively.

## Highlights

• Efficient and rapid platform to integrate DNA without size limits into the genome
• Excision step leaves minimal scarring at the modified locus after the procedure
• Technique expedites generating and evaluating multi-parameter reporter hiPSC lines
• It also facilitates the simultaneous generation of panels of disease variant hiPSCs

## Motivation

It is problematic to insert DNA fragments larger than ∼3 kb into a specific genomic locus in any mammalian cell, including hiPSCs. To address this, we have developed a platform that facilitates such site-specific insertion or replacement of genomic fragments with efficiencies that permit targeting of multiple constructs simultaneously. Compared with other approaches, STRAIGHT-IN demonstrably has virtually no restriction on the size of the DNA that can be inserted while maintaining precise control over modifications to the genome; it can thus significantly improve throughput in generating genetically modified cell lines.

## Graphical abstract

**Figure:**
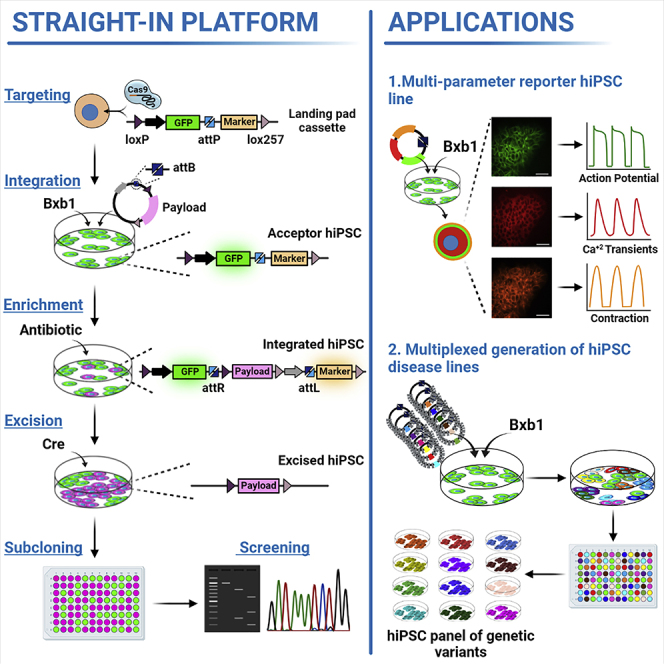

## Teaser

Blanch-Asensio et al. present a method to integrate large DNA payloads into specific genomic sites of hPSCs. This procedure offers a precise and rapid approach to generate genetically matched panels of hPSC lines with applications in research areas such as disease modeling and synthetic biology.

## Introduction

Human pluripotent stem cells (hPSCs) have tremendous potential for advancing the understanding of human development and disease. To realize this, methods for efficient targeted genetic modification are crucial. Endonuclease-based gene-editing systems (i.e., CRISPR-Cas9) have made it significantly easier to perform small-scale genomic modifications in hPSCs. However, strategies to insert multi-kilobase (kb) payloads that consist of several transgenes or a genomic fragment, for example, are limited, since the efficiency of targeting mediated by homology-directed repair decreases significantly with increasing insert size (Byrne et al., 2015; Zhang et al., 2021).

Site-specific recombinases (SSRs) are useful tools for performing difficult genome engineering tasks (e.g., insertion, deletion, or inversion of large DNA segments) in cultured human cells (Turan et al., 2013). SSRs are divided into two families based on the identity of the nucleophilic active site amino acid residue. Phage-derived serine recombinases, which include Bxb1 and φC31 integrases, mediate unidirectional recombination between the enzyme’s unique phage and bacterial attachment sites (*attP* and *attB*, respectively), thereby enabling irreversible integration of transgenes into the genome (Brown et al., 2011). For instance, payloads of up to 33 kb can be efficiently inserted using Bxb1 in Chinese hamster ovary (CHO) cells (Gaidukov et al., 2018). Tyrosine recombinases (e.g., Cre or FLP recombinases) can also integrate DNA fragments (Brosh et al., 2021), although their integration efficiencies are relatively lower and the event reversible (Turan et al., 2013). However, they are effective at excising cassettes flanked by directly repeated recombination sites, leading to their widespread use in conditionally knocking out genes, removing selection cassettes following targeting and in lineage tracing studies (Davis et al., 2008; Skarnes et al., 2011).

Despite both classes of SSRs being used to genetically modify hPSCs (Chaudhari et al., 2020; Du et al., 2009; Ordovás et al., 2015; Zhu et al., 2014), efficient methods to perform targeted integrations of large DNA payloads (>10 kb) that are also suitable for multiplex assays are still lacking. Cassette exchange strategies, whereby a previously targeted landing pad (LP) cassette containing SSR recognition/attachment sites is used to insert transgenes flanked by corresponding sequences, have been developed for repeated modifications of hPSCs or differentiated progenitor cells (Lv et al., 2018; Pei et al., 2015; Zhu et al., 2014). However, the largest payload reported to be inserted was ∼7 kb (Zhu et al., 2014). Alternatively, φC31 and λ integrases can introduce larger inserts (up to ∼20 kb) but only into pseudo-*attP* sites already present in the human genome, thus preventing selection of the target site (Chaudhari et al., 2020; Farruggio et al., 2017; Liu et al., 2009). Furthermore, locus-specific silencing of the transgenes occurred in some instances (Farruggio et al., 2017).

Here, we have coalesced the advantages of both classes of SSRs to develop a platform, which we term STRAIGHT-IN (for serine and tyrosine recombinase-assisted integration of genes for high-throughput investigation). STRAIGHT-IN enables the targeted integration or substitution of large genomic fragments into human induced PSCs (hiPSCs) while leaving minimal traces (<300 bp) of plasmid backbone DNA sequences in the locus. We demonstrate how the platform can be applied to construct synthetic genetic circuits by generating a cell line containing multiple genetic reporters (∼14 kb payload) to assess excitation-contraction coupling in hiPSC-derived cardiomyocytes (hiPSC-CMs). Furthermore, we showcase the ability of STRAIGHT-IN to support multiplex genetic assays by simultaneously generating a library of genetically matched hiPSC lines carrying heterozygous mutations in the gene *KCNH2*, which can result in various cardiac arrhythmia syndromes in patients (Chen et al., 2016). We confirm that the hiPSC-CMs for the *KCNH2* variant A561T reflected the expected electrophysiological disease phenotype. Overall, these results highlight the possibilities offered by STRAIGHT-IN for expanding the range of biological questions that can be investigated using hiPSCs in a high-throughput manner.

## Results

### hiPSC acceptor lines for Bxb1- and φC31-mediated integration

For targeted integration of large genomic fragments into hiPSCs, we first generated acceptor lines that contained a LP cassette. The LP cassettes for both Bxb1 and φC31 were similarly designed and included a constitutive promoter (phosphoglycerate kinase [pGK] promoter) driving expression of a fluorescent protein, an *attP* site recognized by the corresponding serine recombinase, and an antibiotic positive selection marker without an ATG initiation codon. These cassettes were also flanked by heterotypic recognition sites for either Cre or Flp recombinase to enable their excision downstream.

**Figure 1:**
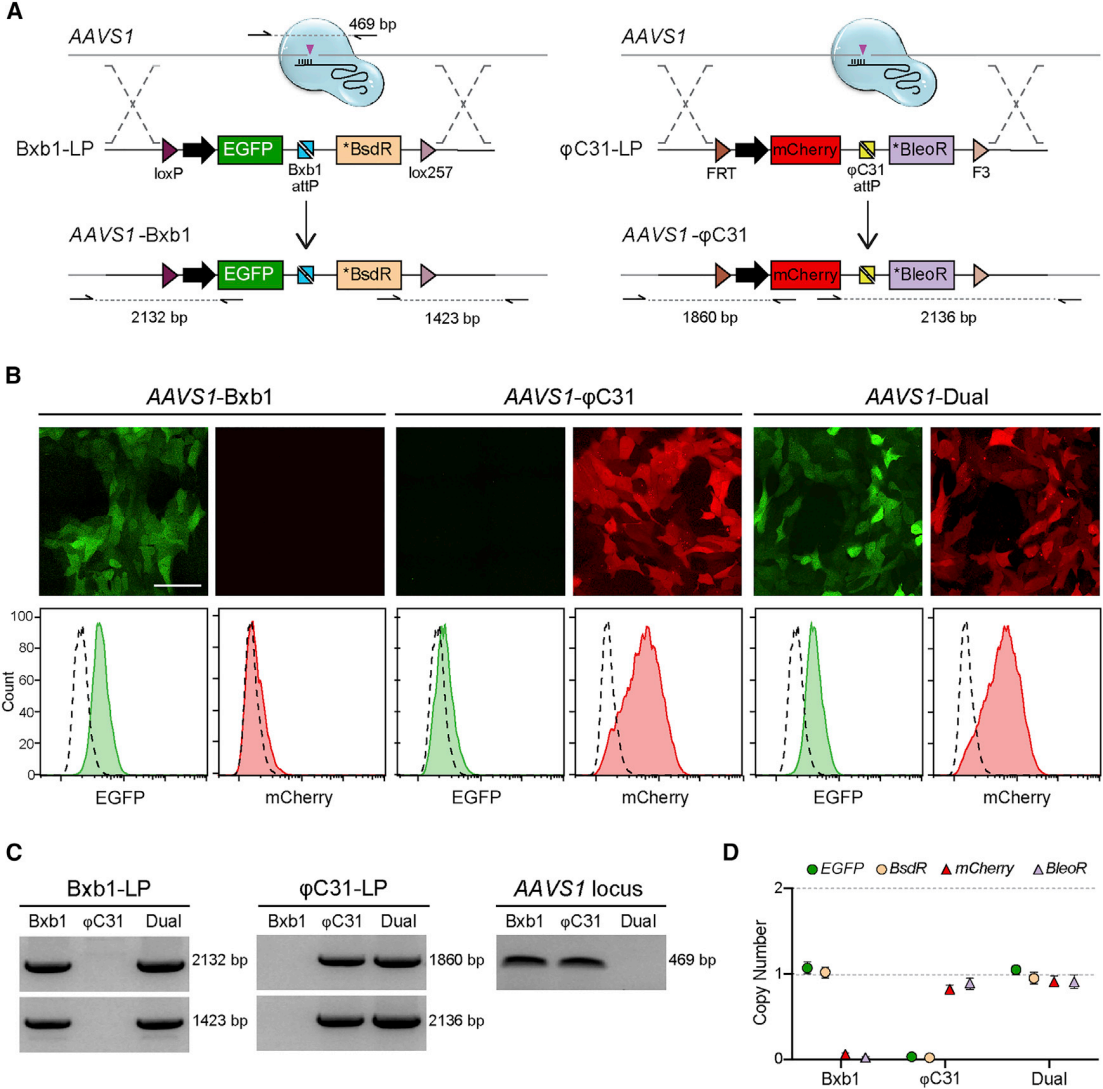
hiPSC acceptor lines for Bxb1- and φC31-mediated integration (A) Schematic for the targeting of the Bxb1- and φC3-landing pad (LP) cassettes to the *PPP1R12C* locus (*AAVS1*). The large black arrows represent the pGK promoter driving constitutive expression of the fluorescent reporter gene EGFP or mCherry. The asterisk (∗) indicates that the antibiotic positive selection marker (blasticidin S deaminase [BsdR] or bleomycin resistance [BleoR]) lacks an ATG initiation codon and so is not expressed in the resulting targeted hiPSCs. Purple triangles indicate the sites of CRISPR-Cas9-induced double-strand DNA breaks. Half arrows indicate primer binding sites with dotted lines representing the resulting PCR amplicons. (B) Fluorescence images (top) and flow cytometry plots (bottom) indicating the expression of the EGFP and mCherry reporters in the resulting targeted *AAVS1*-acceptor hiPSC lines. Scale bar, 50 μm. (C) PCR amplification, using primer pairs indicated in (A), of genomic DNA confirming targeting of the LP cassettes in the resulting *AAVS1*-acceptor hiPSC lines. The LP cassette was targeted to only one allele in the *AAVS1*-Bxb1 and *AAVS1*-φC31 hiPSCs, while both alleles were targeted in the *AAVS1*-Dual hiPSCs (right panel). (D) ddPCR confirming the *AAVS1*-acceptor hiPSC lines had either 0 or 1 copy of each LP cassette inserted into the genomic DNA. Error bars represent Poisson 95% confidence interval (CI). See also Figure S1.

The *AAVS1* locus can support stable, long-term expression of introduced transgenes, including in differentiated hiPSC derivatives such as hiPSC-CMs (Sun et al., 2020). We initially targeted a single copy of each LP cassette to *AAVS1*, generating the hiPSC acceptor lines *AAVS*1-Bxb1 and *AAVS1*-φC31 (Figure 1A). Since each LP construct was comprised of unique components, we also generated an hiPSC line that was biallelically targeted with both LPs (*AAVS1*-Dual), thereby providing a cell line containing an orthogonal pair of target sites that do not cross-react. Fluorescent reporters facilitated the isolation of clonal hiPSCs expressing either GFP (*AAVS1*-Bxb1), mCherry (*AAVS1*-φC31), or both in the case of the *AAVS1*-Dual acceptor line (Figure 1B). Screening and genotyping PCR confirmed the LP cassettes were correctly targeted to the *AAVS1* locus for each of the hiPSC acceptor lines (Figures 1C, S1A, and S1B). In addition, ddPCR for the genes *EGFP*, *mCherry*, *BsdR*, and *BleoR* verified only a single integration of each LP in the respective acceptor lines (Figure 1D).

### Bxb1 mediates effective integration of DNA into hiPSCs without size restrictions

**Figure 2:**
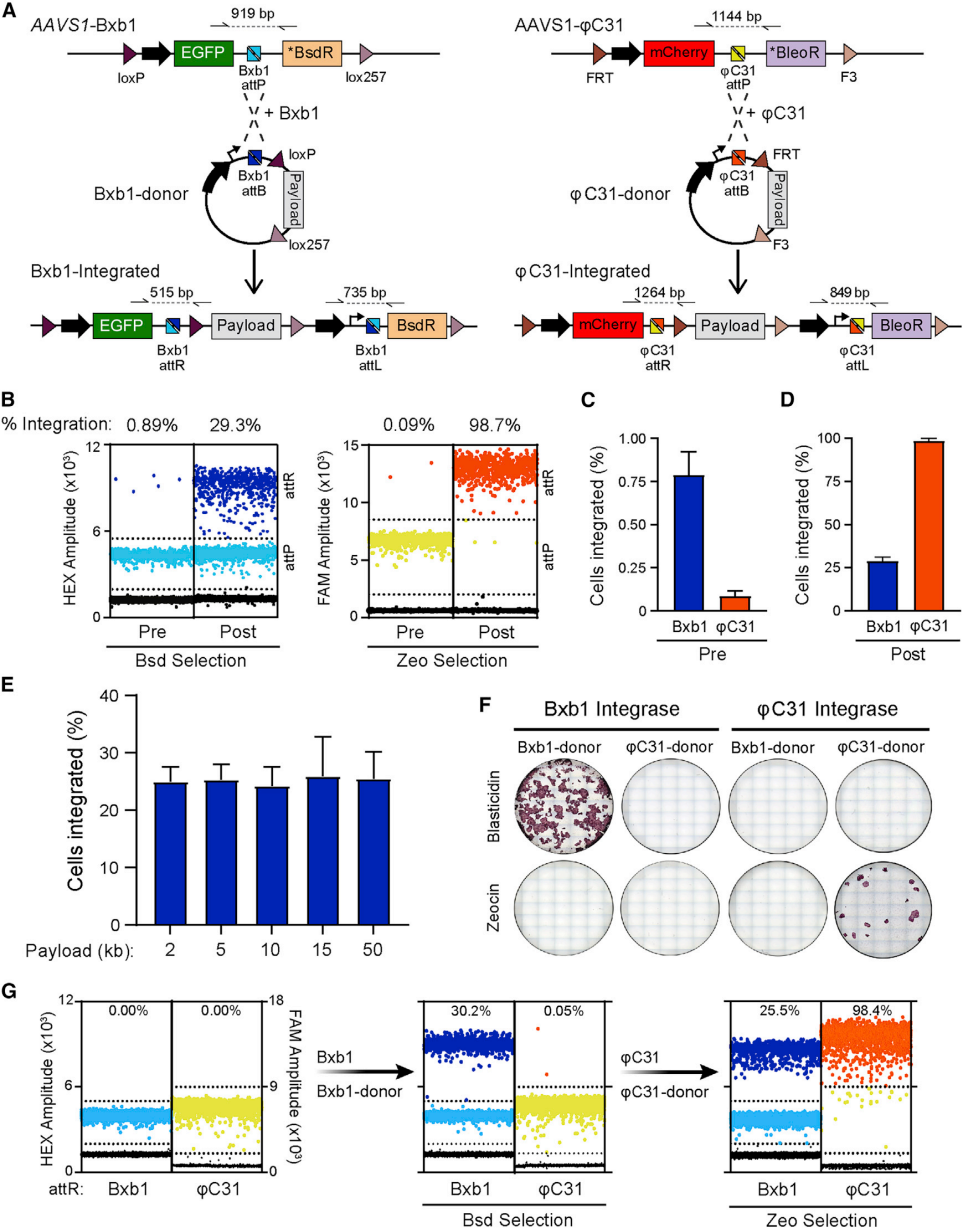
Targeted integration of DNA payloads into hiPSC acceptor lines (A) Schematic for integrating donor constructs by either Bxb1 (left) or φC31 (right) integrases into the corresponding LP cassette targeted to *AAVS1*. Correct integration of the donor construct results in expression of the antibiotic positive selection marker present in the LP cassette. The large black arrows represent constitutive promoters, while “payload” indicates the region in the donor construct where DNA sequences to integrate are inserted. Half arrows indicate primer binding sites with dotted lines representing the resulting PCR amplicons. (B) Representative ddPCR dot plots indicating the integration of either the empty Bxb1 (dark blue) or φC3 (orange) donor vector into *AAVS1-*Dual hiPSCs both before (pre) and after (post) antibiotic enrichment. Dots represent droplets containing the indicated sequence (attR or attP), while the percentages denote the calculated integration efficiency. (C and D) Calculated mean integration efficiency of the Bxb1 and φC3 donor vectors both before (C) and after (D) antibiotic enrichment from 3 transfections. Error bars represent ± SEM. (E) Average percentage of hiPSCs that integrated donor constructs with payloads ranging from 2 to 50 kb into the Bxb1-LP following one round of blasticidin selection. The amount of the DNA delivered into the cells was adjusted based on the size of the plasmid. n = 3 or 4 transfections; error bars represent ± SEM. (F) Alkaline phosphatase staining of hiPSCs following enrichment of cells transfected with plasmids expressing either Bxb1 or φC31 integrase as well as the Bxb1 or φC31 donor vectors demonstrating that integration is both integrase- and selection cassette-specific. (G) ddPCR dot plots demonstrating the sequential integration and enrichment of the Bxb1 (dark blue) and φC31 (orange) donors into *AAVS1-*Dual hiPSCs. Dots represent droplets containing the indicated sequence (attR or attP), while the percentages denote the calculated integration efficiency. See also Figures S1–S3.

To evaluate the efficiency and specificity of the system for exogenous DNA integration, donor vectors specific for both Bxb1- and φC31-LPs were developed (Figures S1C–S1E) and co-transfected with a plasmid expressing either Bxb1 or φC31 into *AAVS1*-Dual. These donor vectors consisted of a constitutive promoter (elongation factor-1 alpha [EF1α]) preceding an ATG initiation codon and the *attB* sequence recognized by either Bxb1 or φC31. Correct integration of the construct into the respective LP would form two new recombination sites (*attR* and *attL*), as well as place the promoter and start codon in the donor vector upstream and in frame with the antibiotic positive selection marker, thereby enabling enrichment of correctly integrated clones by either blasticidin (Bxb1-LP) or zeocin (φC31-LP) selection (Figure 2A).

To detect and quantify the proportion of cells that had integrated the donor vector, ddPCR assays were developed that distinguished both integrated and non-integrated cells (Figure S2). This assay indicated that Bxb1 was ∼10-fold more efficient at mediating the integration of the donor vector than φC31 (Figures 2B and 2C). Additionally, only the integration event mediated by Bxb1 could be detected by diagnostic PCR prior to antibiotic selection (Figure S3A). Although non-integrated cells were more sensitive to zeocin selection than blasticidin (Figure 2D), the integration efficiency of the recombinase is expected to be a more critical step when performing multiplex targeting as this will increase the likelihood that each of the different payloads is integrated. Furthermore, enrichment with blasticidin selection could be improved by maintaining the hiPSCs with the antibiotic for a longer period, thus resulting in ∼80% of the cells having integrated the donor construct (Figure S3B). Therefore, we focused on further evaluating the capabilities of Bxb1 to perform targeted integrations of large DNA payloads into hiPSCs. To determine whether there was a limit on the size of the donor construct that could be integrated, a series of donor vectors with DNA payloads varying in size from ∼2 to ∼50 kb were co-transfected with Bxb1. Approximately 25% of the cells following enrichment had the payload integrated (Figures 2E and S3C), suggesting that overall targeting frequency was independent of size.

The specificity of the integrases to their target sequences was also evident, with no colonies obtained when we mismatched the integrase with the *attB* donor vector (Figure 2F). This also provides the possibility to integrate different payloads into the same hiPSCs. To demonstrate this, we integrated both the Bxb1 and φC31 donors into the *AAVS1*-Dual acceptor line. Following blasticidin and zeocin selection, and mirroring the integration efficiencies observed with each of the recombinases individually, ∼25% of the hiPSCs had integrated the Bxb1 donor, with almost all of these cells also integrating the φC31 donor (Figure 2G).

**Figure 3:**
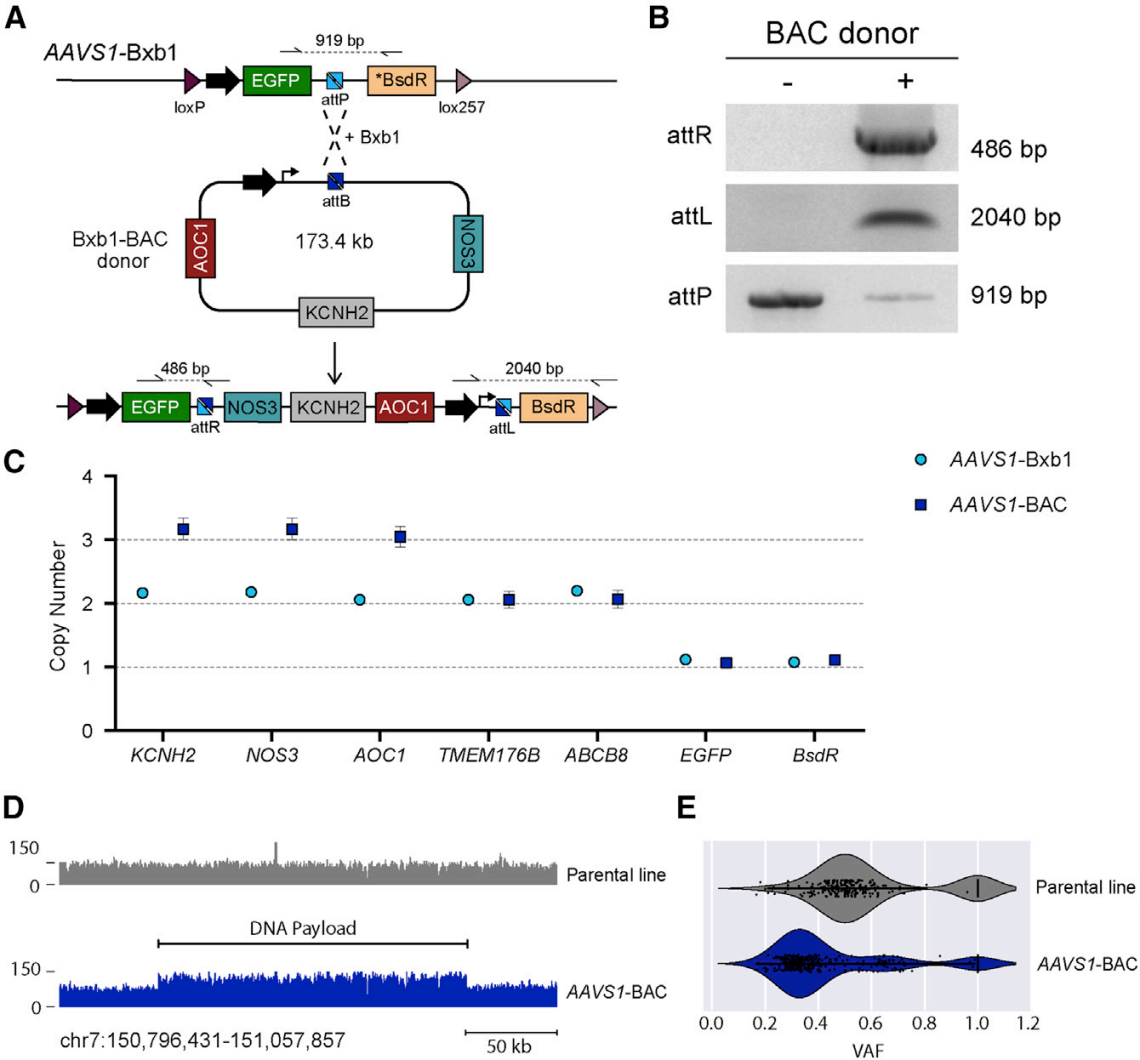
Targeted integration of BAC vectors into hiPSC acceptor lines (A) Schematic for integrating into *AAVS1*-Bxb1 hiPSCs the Bxb1-BAC donor that contains an ∼160 kb genomic region from chromosome 7, which includes the genes *KCNH2*, *NOS3*, and *AOC1.* Half arrows indicate primer binding sites, with dotted lines representing the resulting PCR amplicons. (B) PCR amplification, using primer pairs indicated in (A), of genomic DNA confirming integration of the Bxb1-BAC donor vector into a subset of the *AAVS1-*Bxb1 hiPSCs. The “−” and “+” symbols indicate before and after blasticidin selection, respectively. (C) ddPCR confirming that a hiPSC clone with the Bxb1-BAC donor vector integrated (*AAVS1*-BAC) contained 3 copies of *KCNH2*, *NOS3*, and *AOC1.* The non-integrated *AAVS1*-Bxb1 hiPSC line retained 2 copies of each. Both lines contained two copies of the flanking genes *TMEM176B* and *ABCB8* and a single copy of the LP cassette transgenes *EGFP* and *BsdR.* Error bars represent Poisson 95% CI. (D) WGS of *AAVS1*-BAC hiPSCs showed an increase in the number of sequence reads over a ∼160 kb region on chromosome 7, which includes *KCNH2*, *NOS3*, and *AOC1*, indicating a gain in copies of this region. (E) VAF analysis of the ∼160 kb genomic region confirmed a change in the frequency of heterozygous variants from 0.5 in the parental hiPSC line to ∼0.33 in *AAVS1*-BAC hiPSCs, indicating the presence of 3 copies of this genomic region.

We did not detect an upper limit to the length of the payload that could be targeted, with a modified 173 kb bacterial artificial chromosome (BAC) construct also readily integrating into the *AAVS1* locus as confirmed by screening PCR (Figures 3A and 3B). ddPCR established that the resulting hiPSCs acquired an additional copy of the three genes (*KCNH2*, *NOS3*, and *AOC1*) present on the BAC donor while maintaining two copies of the genes *TMEM176B* and *ABCB8* that neighbor this region on chromosome 7 (Figure 3C). To confirm that the entire DNA payload was integrated and that no rearrangements had occurred, we performed whole-genome sequencing (WGS) of the parental line and one of the hiPSC clones containing the integrated BAC construct. Analysis of the mapped sequence reads in the *AAVS1*-BAC hiPSCs showed an increase in the number of reads spanning the entire genomic region that was present in the BAC construct without any indication of additional structural variants (Figure 3D), therefore indicating that the full DNA payload remained intact following integration. The presence of 3 copies for this genomic region was confirmed by variant allele frequency (VAF) analysis, with heterozygous variants clustering at frequencies of either 0.33 or 0.66 in the *AAVS1*-BAC hiPSCs compared with 0.5 in the parental hiPSC line (Figure 3E).

### Cre and FLP efficiently excise auxiliary sequences following donor vector integration

**Figure 4:**
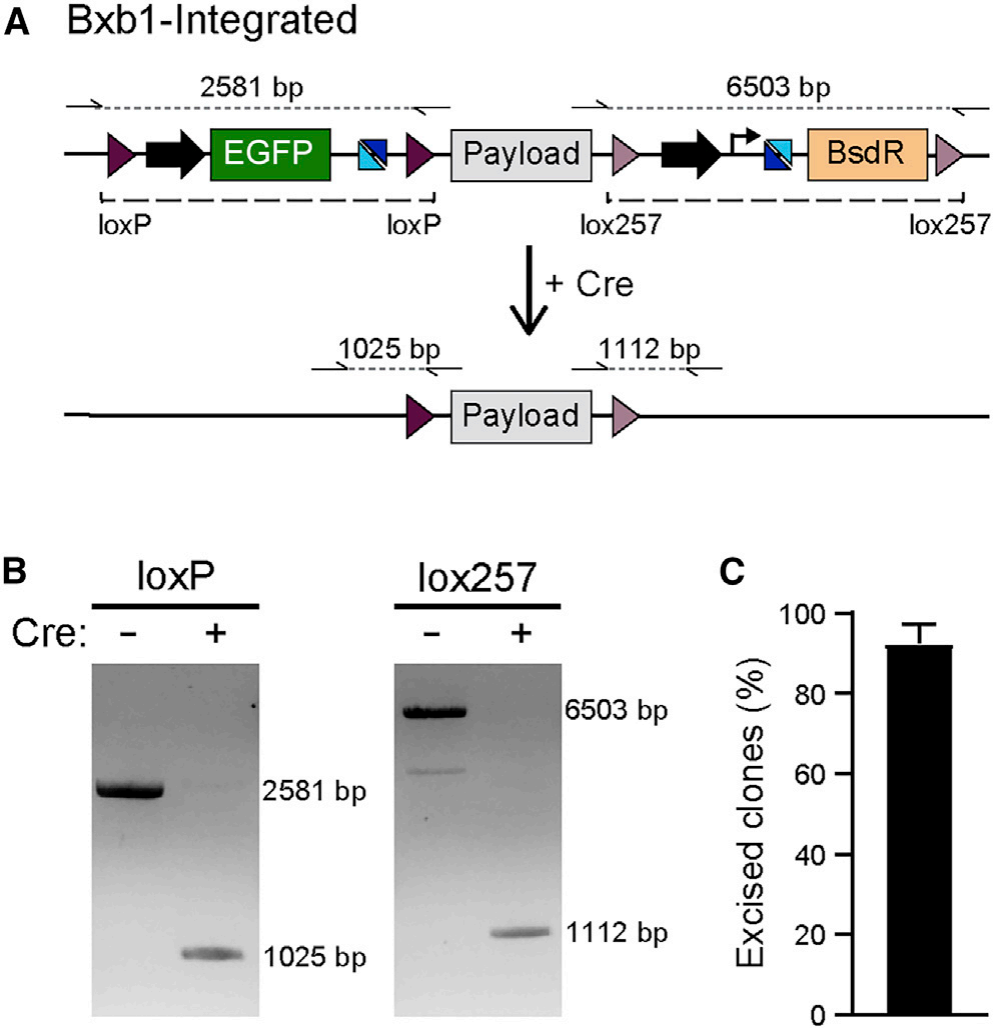
Excision of auxiliary sequences by Cre recombinase (A) Schematic of procedure for excising the positive selection cassettes and vector backbone following integration of the donor vector into the Bxb1-LP. Dashed lines indicate the sequences excised. Half arrows indicate primer binding sites, with dotted lines representing the resulting PCR amplicons. (B) PCR screening using primer pairs indicated in (A), confirming the reduction in amplicon length upon expression of Cre recombinase (+). (C) Quantification of integrated hiPSCs that have excised the auxiliary sequences following Cre expression. n = 5 independent transfections; error bars represent ± SEM. See also Figure S3.

Upon integration, the entire donor vector is integrated into the LP. These auxiliary sequences within the vector backbone can lead to silencing of either the transgenes or neighboring genetic elements (Chen et al., 2004; Pham et al., 1996), with the effect prevented or even reversed if these sequences are subsequently excised from the targeted loci (Davis et al., 2008; Riu et al., 2005). Including *loxP* and *lox257* sequences in both the LP construct and donor vector resulted in both the vector backbone and the majority of the LP cassette being flanked by these recombination sequences following vector integration (Figure 4A). Transiently expressing Cre led to the excision of these auxiliary sequences in >90% of integrated recombinant clones, leaving only the integrated DNA payload plus a single copy of *loxP* and *lox257* at the targeted *AAVS1* locus (Figures 4B and 4C). We also confirmed that FLP recombinase could efficiently excise FRT- and F3-flanked sequences following φC31-mediated integration of donor vectors (Figures S3D–S3F).

Overall, combining Bxb1-mediated integration of DNA vectors with subsequent Cre-mediated excision of the auxiliary sequences resulted in targeted clonal hiPSC lines being generated within 6 weeks, irrespective of the size of the DNA integrated. Furthermore, due to the efficiency of the recombinases combined with drug selection, correctly targeted cells were identified when screening typically <10 clones.

### STRAIGHT-IN expedites evaluating and generating multi-parameter reporter hiPSCs

To demonstrate the utility and rapid adaptability of STRAIGHT-IN, we generated a series of hiPSC lines to initially evaluate individual optogenetic sensors prior to developing a multi-parameter reporter line for assessing excitation-contraction coupling in hiPSC-CMs. These reporters consisted of ASAP2f for assessing the cardiac action potential (AP) (Zhang et al., 2019), jRCaMP1b for measuring cytosolic Ca^2+^ levels (Dana et al., 2016), and a far-red fluorescent reporter fused to a plasma membrane localization signal (Lck-miRFP703) (Chertkova et al., 2020; Shcherbakova et al., 2016) to quantify contraction. To simplify construction of the donor vectors, a modular cloning strategy was employed in which the basic components (e.g., promoter, localization signal, coding DNA sequence [CDS], terminator) were initially constructed in a one-step golden gate cloning reaction (Weber et al., 2011). The resulting expression cassettes could then be assembled either individually or as a complex multi-unit circuit into a modified donor vector.

Each reporter was individually integrated into the *AAVS1-*Bxb1 hiPSCs and auxiliary sequences excised, with targeted clones confirmed by genotyping PCR (Figures S4A and S4B). Fluorescence imaging and flow cytometry established that the reporters were constitutively expressed either in the cell membrane (ASAP2f, miRFP703) or the cytosol (jRCaMP1b) of hiPSCs (Figure S4C). All three hiPSC lines differentiated into hiPSC-CMs, which displayed characteristic sarcomeric structures, as evidenced by α-actinin staining (Figure S4D), and the expression of the reporters was maintained and localized to the expected subcellular regions (Figure S4E). Furthermore, these fluorescent sensors facilitated the assessment of APs, Ca^2+^ transients, and contraction in hiPSC-CMs (Figure S4F). The hiPSC-CMs expressing ASAP2f showed the expected periodic changes in fluorescence intensity, with a reduction detected in the depolarization phase, followed by an increase during membrane repolarization and the diastolic resting phase. Likewise, jRCaMP1b-expressing hiPSC-CMs displayed cyclic changes in fluorescence, with an increase in fluorescence intensity during the systolic rise in intracellular calcium levels, followed by a reduction in fluorescence during relaxation. Finally, contraction could be quantified by measuring the displacement of membrane-localized miRFP703 (Sala et al., 2018).

**Figure 5:**
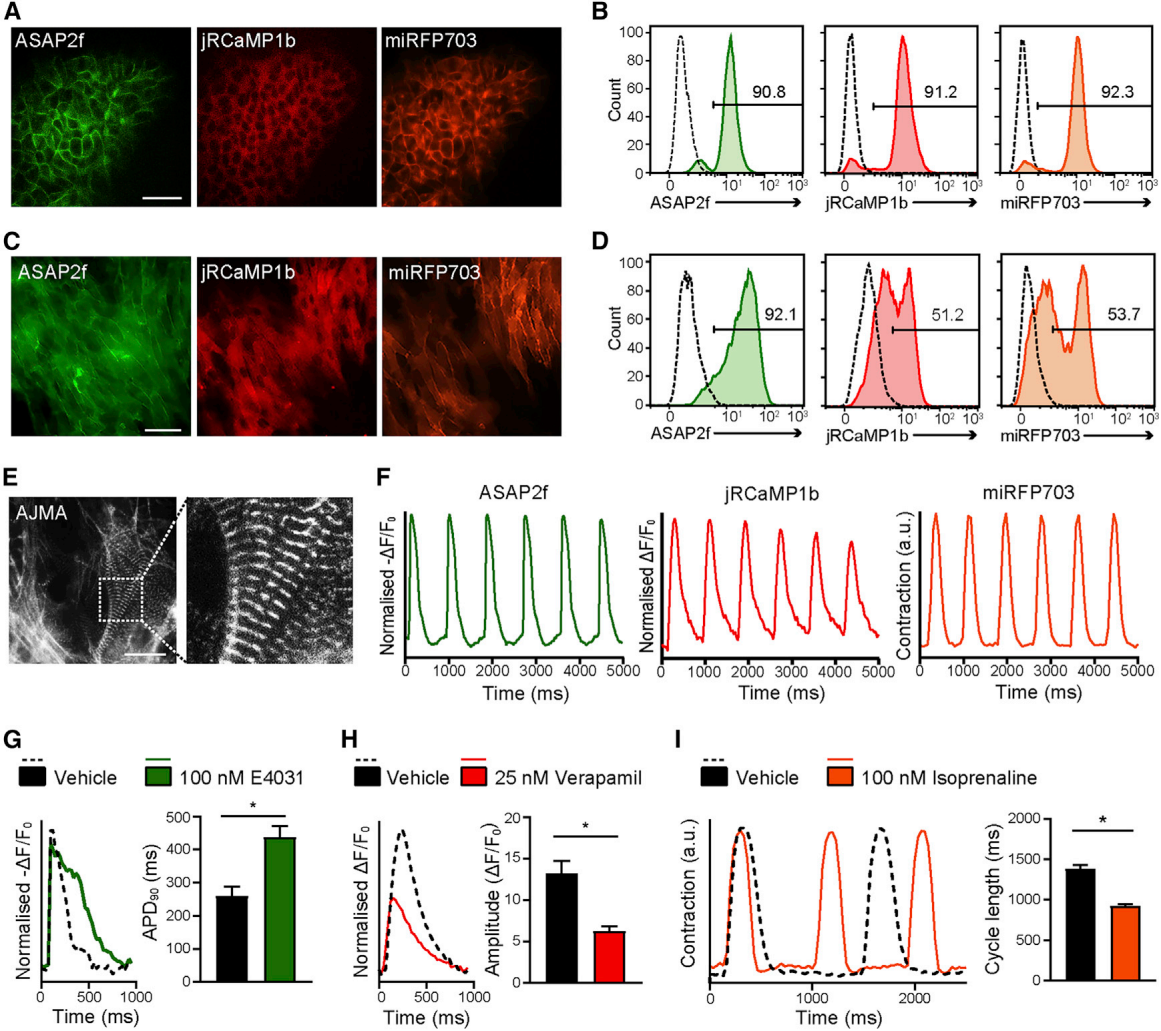
Generation of a multi-parameter reporter hiPSC line using STRAIGHT-IN (A–D) Fluorescence images (A and C) and flow cytometric analysis (B and D) of *AAVS1*-AJMA hiPSCs (A and B) and hiPSC-CMs (C and D) indicating the cellular localization and expression level of each of the reporters integrated. Scale bars, 75 μm. Numbers in histograms indicate the percentage of cells positive for the specified reporter; dotted lines indicate negative control. (E) Immunofluorescence image of the cardiac sarcomeric protein α-actinin in *AAVS1-AJMA* hiPSC-CMs. Image on the right is a magnification of the region within the dotted boxes. Scale bar, 25 μm. (F) Representative time plots of baseline-normalized fluorescence signals from *AAVS1*-AJMA hiPSC-CMs stimulated at 1.2 Hz. Changes in the fluorescence of ASAP2f (left) and jRCaMP1b (middle) reflect the action potential and cytosolic Ca^2+^ transients, respectively, while displacement of the miRFP703 (right) fluorescence signal indicates contraction dynamics. (G–I) Representative AP (G, left), cytosolic Ca^2+^ (H, left), and contraction (I, left) transients of *AAVS1*-AJMA hiPSC-CMs treated with vehicle (0.1% DMSO) or indicated compounds, together with the resulting average APD_90_ (G, right), Ca^2+^ peak amplitude (H, right), and contraction cycle length (I, right) values. n = 4 (vehicle) and n = 5 (drug) treated samples; error bars ± SEM; ∗p < 0.01 (unpaired t test). See also Figure S4.

Accordingly, we constructed a donor vector comprising all three reporters plus an additional copy of ASAP2f to increase its expression in hiPSC-CMs (Figure S4G). Using STRAIGHT-IN, we integrated the multi-reporter construct into the *AAVS1-*Bxb1 hiPSCs (∼35% efficiency) and excised the LP cassette and vector backbone (Figures S4H and S4I). The resulting hiPSC line (*AAVS1*-AJMA) contained a ∼14 kb DNA payload and co-expressed all 3 reporters in >90% of the cells, with expression also maintained in later passage hiPSCs (Figure 5A, 5B, and S4J). All 3 reporters were also expressed in hiPSC-CMs, although with some silencing of jRCaMP1b and Lck-miRFP703 possibly due to the absence of insulator sequences in the multi-reporter construct (Sharma et al., 2012) (Figures 5C–5E, and S4G). Nevertheless, intracellular Ca^2+^ signaling and contraction still could be assessed using these sensors (Figure 5F).

Furthermore, we evaluated the ability of *AAVS1*-AJMA CMs to detect changes in APs, intracellular Ca^2+^ transients, and contraction profiles for cardiac safety pharmacology applications. For electrophysiological responses, the hiPSC-CMs were treated with a specific hERG channel blocker, E-4031. Compared with the vehicle control (0.1% DMSO), significant prolongation in AP duration at 90% repolarization (APD_90_) was observed following the addition of E-4031 (Figure 5G). Similarly, changes in Ca^2+^ handling were detected in the presence of verapamil, a multi-channel blocking compound. Consistent with its mechanism as an L-type Ca^2+^ channel blocker, a decrease in Ca^2+^ transient amplitude was observed (Figure 5H). Finally, addition of the beta-adrenergic agonist isoprenaline resulted in a shortening of the contraction duration of the hiPSC-CMs and a significant decrease in mean cycle length (Figure 5I).

Together, these results demonstrate how STRAIGHT-IN, combined with adaptations to the donor vector for modular cloning and the development of a component library, can be applied to rapidly construct and integrate synthetic genetic circuits, thus permitting the comparison of various transgenes under the same chromosomal environment to exclude position effects. The stable integration of these reporters into a genomic safe-harbor locus minimizes the unpredicted consequences of viral-based approaches and offers the possibility to repeatedly measure the hiPSC-CMs over multiple time points, for example to monitor the maturation of the cells or to evaluate chronic pharmacological responses (Karbassi et al., 2020; De Korte et al., 2020).

### STRAIGHT-IN facilitates the simultaneous generation of a panel of disease variant hiPSCs

Lastly, we investigated whether the platform supported multiplex genetic assays by simultaneously generating a library of hiPSC lines carrying heterozygous mutations in *KCNH2*. Mutations in *KCNH2*, which encodes the hERG ion channel, can cause various cardiac arrhythmia syndromes including long QT syndrome type 2 (LQT2), short QT type 1, and Brugada syndrome (Chen et al., 2016). However, there are also rare *KCNH2* variants that do not affect the functionality of the encoded ion channel (Ng et al., 2020). Defining whether these variants are disease causing or innocuous is critical for determining the best course of action for treating the patient (Giudicessi et al., 2018).

**Figure 6:**
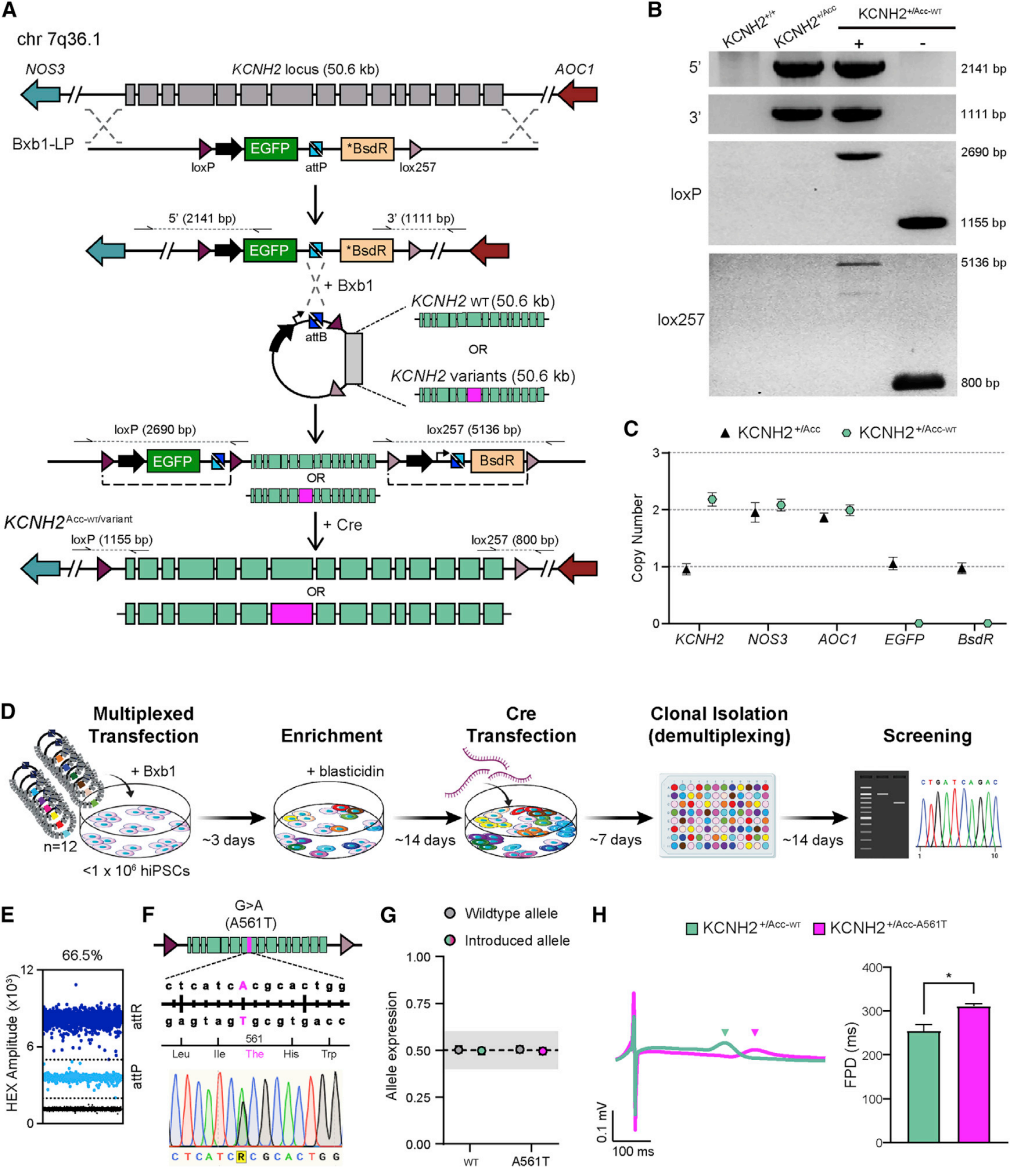
Simultaneous generation of a panel of *KCNH2*-variant hiPSC lines using STRAIGHT-IN (A) Schematic of STRAIGHT-IN procedure to perform targeted heterozygous modifications to a 50.6 kb genomic region on chromosome 7, which includes *KCNH2*. Half arrows indicate primer binding sites, with dotted lines representing the resulting PCR-generated amplicons. Dashed lines indicate the sequences excised by Cre recombinase. (B) PCR products amplified with the corresponding primer pairs indicated in (A), confirming targeting of Bxb1-LP to *KCNH2* (*KCNH2*^+/Acc^), and subsequent reintroduction of wildtype *KCNH2* (*KCNH2*^+/Acc-^^WT^). Auxiliary sequences detected upon integration (+) were excised following Cre expression (−). The sizes of the amplicons are indicated. (C) ddPCR confirming that *KCNH2*^+/Acc^ and *KCNH2*^+/Acc-WT^ hiPSCs contained the expected number of copies of the genomic genes *KCNH2* (1 and 2 copies, respectively), *NOS3* and *AOC1* (both 2 copies), and the Bxb1-LP cassette transgenes *EGFP* and *BsdR* (1 and 0 copies, respectively). Error bars represent Poisson 95% CI. (D) Schematic of the STRAIGHT-IN procedure for simultaneously generating and identifying isogenic hiPSC clones for 12 different *KCNH2* variants, along with the approximate time required for each step. (E) Dot plot of *KCNH2*^*+/Acc*^ hiPSCs transfected with the 12 *KCNH2* variant donor vectors. Dots represent droplets containing the indicated sequence (attR or attP), while the percentage denotes the calculated integration efficiency. (F) Overview of the genomic sequence and location within *KCNH2* of the introduced variant A561T, and sequence analysis from one of the resulting *KCNH2*^*+/Acc-A561T*^ hiPSC clones indicating the heterozygous introduction of c.G1681A. (G) Quantification of *KCNH2* expression in *KCNH2*^*+/Acc-WT*^ and *KCNH2*^*+/Acc-A561T*^ hiPSC-CMs confirming balanced allelic expression from the wild-type (WT) and reintroduced alleles. The shaded area indicates the region close to 0.5 (0.4–0.6). Error bars represent Poisson 95% CI. (H) Representative averaged field potential (FP) traces (left) and averaged FP duration (FPD) values (right) of *KCNH2*^+/Acc-WT^ and *KCNH2*^+/Acc-A561T^ hiPSC-CMs paced at 1.25 Hz. Colored arrowheads indicate the respective repolarization peak for each line. n = 23 (*KCNH2*^+/Acc-WT^) and 29 (*KCNH2*^+/Acc-A561T^) recordings; error bars represent ±SEM, and ∗p < 0.0001 (unpaired t test). See also Figures S5 and S6.

As there appears to be no limit on the length of the DNA sequence that can be inserted using STRAIGHT-IN, we opted to replace the entire *KCNH2* genomic locus (50.6 kb) on one allele with the Bxb1-LP (Figure 6A). This meant that the introduced heterozygous *KCNH2* variants would be in an almost identical genomic context to that in affected individuals. Genotyping and ddPCR confirmed that the resulting hiPSC line (*KCNH2*^+/Acc^) was correctly targeted, contained a single integration of the Bxb1-LP, and was monoallelic for *KCNH2* while retaining two copies of the flanking genes, *NOS3* and *AOC1* (Figures 6B and 6C). Furthermore, WGS and VAF analysis corroborated that the expected deletion of one of the *KCNH2* alleles had occurred (Figures S5A and S5B).

We initially reintroduced the *KCNH2* wild-type sequence into the *KCNH2*^+/Acc^ hiPSC line as confirmed by PCR screening (Figure 6B). From 59 clones screened, 18 (∼30%) had undergone integration and excision, showing similar efficiency to that observed for the *AAVS1* locus. ddPCR confirmed for one of the hiPSC clones (*KCNH2*^+/Acc-WT^) that it was now biallelic for *KCNH2,* while both *EGFP* and *BsdR* were absent (Figure 6C). Furthermore, *KCNH2*^+/Acc-WT^ was karyotypically normal and differentiated into hiPSC-CMs with similar efficiency as the original wild-type hiPSCs (KCNH2^+/+^) (Figures S5C–S5E). Additionally, no differences in cardiac field potential duration (FPD) were observed between the two lines, indicating that the electrophysiological activity of the hiPSC-CMs was also unaffected by the STRAIGHT-IN procedure (Figures S5F).

Based on this, we constructed donor vectors for 12 *KCNH2* variants identified in exon 7 on ClinVar (Table S1), which were subsequently pooled and transfected along with Bxb1 into <1 × 10^6^ KCNH2^+/Acc^ hiPSCs (Figure 6D). Modifications to the enrichment step improved the proportion of recombined hiPSCs to 66.5% (Figure 6E), with all 12 variants detected by ddPCR within this mixed population (Figure S6A). Following Cre-mediated excision of the auxiliary sequences and single-cell deposition, 11 out of the 12 variants were identified by Sanger sequencing from 208 subclones screened by PCR (Figure S6A), with the entire procedure taking ∼2 months. VAF analysis of the KCNH2 genomic sequence reintroduced as well as the genomic regions flanking it confirmed no rearrangements had occurred in the *KCNH2*^+/Acc-WT^ hiPSCs or in clones for 10 of the 11 recovered variants (Figure S6B). For the *KCNH2* variant T618S (*KCNH2*^+/Acc-T618S^), a cluster for heterozygous variants was no longer observed at 0.5 within the reintegrated sequence, suggesting a potential deletion or rearrangement occurred during cloning or when performing STRAIGHT-IN.

We further characterized an hiPSC clone identified as being heterozygous for the *KCNH2* variant A561T (*KCNH2*^+/Acc-A561T^) (Figure 6F). ddPCR verified STRAIGHT-IN had occurred as expected and that the hiPSCs had a normal karyotype (Figures S6C and S6D). Moreover, both *KCNH2*^+/Acc-WT^ and *KCNH2*^+/Acc-A561T^ hiPSC-CMs showed balanced allelic expression of the *KCNH2* transcript (Figure 6G), confirming that introducing the *KCNH2* variants using STRAIGHT-IN did not alter gene expression. hiPSC-CMs that carry the *KCNH2* mutation A561T exhibit a prolonged FPD (Brandão et al., 2020; Matsa et al., 2011), reflecting the electrophysiological characteristics of LQT2. The resulting *KCNH2*^+/Acc-A561T^ hiPSC-CMs had a significantly prolonged FPD compared with *KCNH2*^+/Acc-WT^ hiPSC-CMs (Figure 6H), thereby confirming that *KCNH2* variant models generated using the STRAIGHT-IN procedure likewise can exhibit the expected disease phenotype.

Overall, these results demonstrate how STRAIGHT-IN also can be used as a high-throughput method to multiplex and simultaneously insert potentially hundreds of different disease-linked variants into a control hiPSC line.

## Discussion

In this study, we present STRAIGHT-IN, an efficient and modular platform for targeted genomic integration of DNA payloads into hiPSCs. The workflow of STRAIGHT-IN consists of three steps: (1) targeting a LP cassette to the locus of interest, (2) integrating a donor vector encoding the DNA payload into the LP cassette via a serine recombinase, and (3) expressing a tyrosine recombinase to excise the majority of the accessory exogenous DNA sequences. The resulting hiPSC lines contain the targeted DNA payload, with minimal traces of unrequired sequences remaining in the modified locus.

With STRAIGHT-IN, we successfully targeted two separate loci in hiPSCs with similar efficiencies, indicating the procedure is not locus dependent and could likely be used to modify any locus of interest. Additionally, two distinct LP cassettes and donor constructs were designed that utilized different serine and tyrosine recombinases. Although we have focused on the capabilities of Bxb1-mediated recombination, the specificity of the recombinases also enables dual modification of a cell line using STRAIGHT-IN, thereby further broadening the flexibility of the system. Additional modifications to the LP cassettes, such as the inclusion of mutant *attP* and *attB* sites that result in better integration efficiencies (Jusiak et al., 2019) or the replacement of *BsdR* with a marker that supports more efficient positive selection, would likely further improve the utility of the procedure. For example, this could enable biallelic integrations to be performed simultaneously (Ohno et al., 2022).

Our results also suggest that there is no constraint on the size of the DNA payload that can be integrated, overcoming one of the main limitations of other commonly used DNA integration systems (e.g., viral vectors, programmable nuclease knockin). To date, the largest payloads reported to be integrated into a single artificial LP in hiPSCs were <10 kb (Zhu et al., 2014). Here, we were readily able to integrate DNA sequences varying between ∼14 and 50 kb in length and even managed to integrate a donor vector containing a 173 kb BAC fragment. Subsequent tyrosine recombinase-mediated excision of the LP cassette and donor vector backbone was also very efficient, with >90% of the integrated cells having these auxiliary sequences excised. This step of the procedure also could be performed by transfecting the recombinase as mRNA, ensuring expression was transient and eliminating the risk of insertional mutagenesis.

For various research and clinical applications, there is an increasing need to precisely and rapidly integrate large DNA fragments (Zhang et al., 2021). STRAIGHT-IN could potentially simplify the generation of cell lines or animal models containing these large and complex genetic circuits. Here, we demonstrate the possibility to quickly prototype synthetic genetic circuits using STRAIGHT-IN. By first individually evaluating each of the genetic reporters for assessing voltage, intracellular Ca^2+^, and contraction in hiPSC-CMs, we concluded that 2 copies of ASAP2f in the multi-reporter construct would be beneficial for clearer detection of voltage changes. Such an approach will likely assist in improving and developing more complicated lineage tracing systems that utilize dynamic DNA barcoding to capture cell fate (McNamara et al., 2022). These have faced technical limitations in part due to the random integration of the molecular “recorders” in the genome and the size constraints of viral delivery systems which can necessitate multiple genetic modification steps (He et al., 2022). Moreover, this platform could be used as a tool for gene therapy and in the assembly of custom-designed mammalian chromosomes (Boeke et al., 2016).

We envision that STRAIGHT-IN will be especially valuable for the large-scale generation of disease panels for precision medicine applications and demonstrate the applicability of the procedure to generate a panel of hiPSC lines with mutations in *KCNH2* simultaneously. Such panels could be used to test the efficacy of pharmacological compounds against individual mutations. For example, nearly 500 mutations in *KCNH2* have been associated with LQT2, with at least 170 of these predicted to cause trafficking defects (Anderson et al., 2014). Orkambi, an approved drug for treating cystic fibrosis in certain patients, was identified as a potential therapeutic for patients with LQT2 caused by trafficking defective variants and is now being clinically evaluated (Schwartz et al., 2019; Wu et al., 2019). However, for some variants, one of the compounds present in Orkambi, lumacaftor, appeared to cause an opposite effect (O’Hare et al., 2020). Therefore, large-scale efficacy studies of the drug using a cohort of *KCNH2* variant hiPSC-CMs will likely be required to advance this further in the clinic.

Another purpose that would benefit from this multiplex approach is classifying rare variants. Large-scale genetic sequencing projects have revealed that rare variants are highly prevalent in the general population. The difficulty in distinguishing pathogenic variants from rare benign variants when performing genetic testing for inherited disorders has resulted in large proportions of patients having variants classified as being of “uncertain significance” and so not clinically actionable. Therefore, platforms that are rapid and do not require an individualized targeting strategy for each variant would be highly advantageous in a diagnostic setting. A previous study investigated whether a dual integrase cassette exchange strategy could generate such a hiPSC panel of *TNNT2* variants (Lv et al., 2018). While heterozygous clones for 14 coding variants were isolated, the reported efficiency was ∼5%, and these lines represented only 12% of the variants introduced. Additionally, the procedure was restricted to integrating a DNA payload of <1 kb, and so only a partial cDNA spanning *TNNT2* exons 6 to 17 could be examined. Not only did this prevent all *TNNT2* CDS variants being investigated but potentially also resulted in the loss of regulatory elements that controlled expression of the variant. Although we modified a different genetic locus, we reported a much higher targeting efficiency (∼34%) with STRAIGHT-IN and could recover >90% of the variants introduced. Furthermore, with our platform, non-coding genomic regions are retained in the integration. This also permits SNPs identified in genome-wide association studies that might influence the disease phenotype to be modified and investigated.

### Limitations of the study

STRAIGHT-IN is most suitable when the same genomic region will be repeatedly modified or when the DNA cargo for targeting exceeds 5 kb. Other targeting approaches, such as those mediated by programmable nucleases, are likely to be more appropriate if only a few independent modifications (e.g., <4) to a single genomic locus are anticipated or if the disease variants being investigated are in separate, distinct loci.

To date, we have only established the platform in human iPSCs; however, we believe that this approach is broadly applicable for use in other cell types such as adult stem cells (Menche and Henner, 2021). In some instances, the LP cassette, which can be easily customized, might require modifying to provide alternative approaches for isolating the integrated clones (for example, via cell surface markers). Likewise, the current method for excising the unrequired sequences flanking the DNA payload following integration results in traces of these sequences remaining in the modified locus (<300 bp). These remaining sequences are beneficial as they simplify screening procedures, and currently, the resulting cell lines are assessed to confirm that the residual auxiliary sequences do not affect expression from the modified locus. However, recent methods developed for scarless excision potentially could enable the complete removal of these sequences (Li et al., 2013; Roberts et al., 2019).

## STAR★Methods

### Key resources table

**Table:** 

REAGENT or RESOURCE	SOURCE	IDENTIFIER
**Antibodies**		

Anti-alpha-actinin (Sarcomeric) antibody (ACTN2)	Sigma-Aldrich	Cat#A7811; RRID: AB_476766
Alexa Fluor 350	ThermoFisher	Cat#A-11045; RRID: AB_2534100
Cardiac Troponin T Antibody, anti-human/mouse/rat, VioBlue®, REAfinity™	Miltenyi Biotec	Cat#130-120-402; RRID:AB_2783891
Cardiac Troponin T Antibody, anti-human/mouse/rat, FITC, REAfinity™	Miltenyi Biotec	Cat#130-119-575; RRID:AB_2751735

**Bacterial and virus strains**		

10-beta Competent *E. coli*	New England Biolabs	Cat#C3019H
Stbl2™ *E. coli*	ThermoFisher	Cat#10268019
NEB® Stable Competent *E. coli*	New England Biolabs	Cat#C3040I
BAC	BACPAC Genomics	RP11-10L20

**Chemicals, peptides, and recombinant proteins**		

X-Gal Solution, ready-to-use	ThermoFisher	Cat#R0941
L-(+)-Arabinose	Sigma–Aldrich	Cat#A3256
DpnI	New England Biolabs	Cat#R0176S
HindIII	New England Biolabs	Cat#R0104S
EcoRI	New England Biolabs	Cat#R0101S
PvuI	New England Biolabs	Cat#R0150S
NsiI	New England Biolabs	Cat#R0127S
Esp3I	New England Biolabs	Cat#R0734S
HaeIII	New England Biolabs	Cat#R0108S
MseI	New England Biolabs	Cat#R0525S
BpiI	ThermoFisher	Cat#ER1011
FastDigest Eco31I	ThermoFisher	Cat#FD0293
T4 DNA Ligase	New England Biolabs	Cat#M0202T
Primocin®	InvivoGen	Cat#ant-pm-05
Blasticidin S hydrochloride	Sigma–Aldrich	Cat#15205
Zeocin™ Selection Reagent	ThermoFisher	Cat#R25001
Puromycin dihydrochloride	Sigma–Aldrich	Cat#P9620
Alkaline phosphatase detection kit	Merck	Cat#SCR004
StemFlex™ Medium	ThermoFisher	Cat#A3349401
Laminin-521	BioLamina	Cat#LN521-02
Matrigel	Corning	Cat#354277
TrypLE Select	ThermoFisher	Cat#A1217701
Accutase® solution	Sigma–Aldrich	Cat#A6964
Lipofectamine™ Stem Transfection Reagent	ThermoFisher	Cat#STEM00003
RevitaCell™ Supplement	ThermoFisher	Cat#A2644501
Bovine Serum Albumin	Bovogen Biologicals Australia	Cat#BSAS05
CHIR99021	Axon Medchem	Cat#Axon 1386
XAV939	Tocris	Cat#3748/10
IWP-L6	AbMole	Cat#M2781
Knockout Serum Replacement	ThermoFisher	Cat#10828028
Dimethyl Sulfoxide	Sigma–Aldrich	Cat#D2650
StemMACS™ Cre Recombinase mRNA	Miltenyi Biotec	Cat#130-101-113
FIX and PERM™ Cell Permeabilization Kit	ThermoFisher	Cat#GAS003
E-4031 dihydrochloride	Tocris	Cat#1808
Verapamil hydrochloride	Tocris	Cat#0654
Isoprenaline hydrochloride	Sigma–Aldrich	Cat#I5627
all-*trans* retinal	Sigma–Aldrich	Cat#R2500
RNAse A	Invitrogen	Cat#8003088

**Critical commercial assays**		

CloneSmart® HCKan Blunt Cloning Kit	Sigma–Aldrich	Cat#LUC407042
Plasmid-Safe™ ATP-Dependent DNase	Biosearch Technologies	Cat#E3101K
NEBuilder® HiFi DNA Assembly Cloning kit	New England Biolabs	Cat#E5520S
NEB® PCR Cloning Kit	New England Biolabs	Cat#E1202S
ddPCR Supermix for Probes (no dUTP)	Bio-Rad	Cat#1863024
QuickExtract™	Biosearch Technologies	Cat#QE905T
High Pure PCR Template Preparation Kit	Roche	Cat#11796828001
EnGen® sgRNA Synthesis Kit	New England Biolabs	Cat#E3322V
PCR product using the Wizard® SV Gel and PCR Clean-Up System kit	Promega	Cat#A9281
NucleoBond® Xtra Midi Kit	Macherey Nagel	Cat#740410.50
NucleoSpin® RNA	Macherey Nagel	Cat#740984.50
DNA-free™ Kit DNase Treatment and Removal Reagents	ThermoFisher	Cat#AM1906
iScript™ cDNA Synthesis Kit	Bio-rad	Cat#1708891

**Deposited data**		

DNA sequencing data	This paper	https://www.ebi.ac.uk/biostudies/arrayexpress/studies/E-MTAB-11971

**Experimental models: Cell lines**		

LUMC0020iCTRL-06 hiPSC line	LUMC hiPSC core facility	RRID: CVCL_ZA25
AAVS1_Bxb1 hiPSC line	This paper	LUMC0020iAAVS-01
AAVS1_φC31 hiPSC line	This paper	LUMC0020iAAVS-02
AAVS1_Dual hiPSC line	This paper	LUMC0020iAAVS-03
AAVS1_ASAP2f hiPSC line	This paper	LUMC0020iAAVS-04
AAVS1_jRCaMP1b hiPSC line	This paper	LUMC0020iAAVS-05
AAVS1_miRFP703 hiPSC line	This paper	LUMC0020iAAVS-06
AAVS1_AJMA hiPSC line	This paper	LUMC0020iAAVS-07
KCNH2^+/Acc^ hiPSC line	This paper	LUMC0020iHERG-17
KCNH2^+/^^WT^ hiPSC line clone D1 KCNH2^+/^^WT^ hiPSC line clone D6 KCNH2^+/^^WT^ hiPSC line clone G9	This paper	LUMC0020iHERG-18 LUMC0020iHERG-19 LUMC0020iHERG-20
KCNH2^+/A561T^ hiPSC line clone D6 KCNH2^+/A561T^ hiPSC line clone E12 KCNH2^+/A561T^ hiPSC line clone F2	This paper	LUMC0020iHERG-21 LUMC0020iHERG-22 LUMC0020iHERG-23

**Oligonucleotides**		

See Tables S2–S4	Integrated DNA Technologies	N/A

**Recombinant DNA**		

MoClo Toolkit	(Weber et al., 2011)	Addgene Kit#1000000044
pAGM4673	(Weber et al., 2011)	Addgene Plasmid#48014
AAVS1_SA_2A_*Neo*_CAG_RTTA3	(Sim et al., 2014)	Addgene Plasmid#60431
pCAG-NLS-HA-Bxb1	(Hermann et al., 2014)	Addgene Plasmid#51271
pCAG-φC31	(Ohtsuka et al., 2015)	Addgene Plasmid#62658
pEFBOS_CreIRESpuro	This paper	Addgene Plasmid#183812
pENTR-eGFP-attP(bxb)-∗BsdR	This paper	Addgene Plasmid#183751
pENTR-mCherry-attP(C31)-∗BleoR	This paper	Addgene Plasmid#183752
AAVS1-Bxb1-LP-TC	This paper	Addgene Plasmid#183754
AAVS1-phiC31-LP-TC	This paper	Addgene Plasmid#183755
Bxb1-ASAP2f	This paper	Addgene Plasmid#183757
Bxb1_jRCaMP1b	This paper	Addgene Plasmid#183758
Bxb1_miRFP703	This paper	Addgene Plasmid#183759
Bxb1_AJMA	This paper	Addgene Plasmid#183760
pBR_attB(C31)_FRT	This paper	Addgene Plasmid#183761
pBR_attB(bxb)_lox	This paper	Addgene Plasmid#183762
pBR_attB(bxb)_ccdB_lox	This paper	Addgene Plasmid#183763
p15_attB(bxb)_lox	This paper	Addgene Plasmid#183764

**Software and algorithms**		

CRISPOR	(Concordet and Haeussler, 2018)	N/A
QuantaSoft™ Analysis Pro	Bio-rad	1.0.596
FlowJo software	FlowJo	v. 10.2
MUSCLEMOTION	(Sala et al., 2018)	N/A
Cardiac Analysis Tool	Axion BioSystems, Inc	v. 3.1.5
GraphPad Prism 8	GraphPad	v. 8.2.0
SnapGene®	SnapGene	v. 6.0.2

**Other**		

Neon Transfection System	ThermoFisher	Cat#MPK5000
BD FACSAria™ III	BD Biosciences	N/A
Leica DMI6000B	Leica Microsystems	N/A
QX200™ Droplet Digital PCR System	Bio-rad	Cat#1864100
EVOS™ M7000 Cell Imaging System	ThermoFisher	Cat#AMF7000
MacsQuant VYB flow cytometer	Miltenyi Biotec	N/A
Maestro Pro multiwell MEA platform	Axion BioSystems, Inc.	N/A
Lumos™ Optical Stimulation System	Axion BioSystems, Inc.	Cat#LUMOS-MEA-96
96 well Lumos MEA plate	Axion BioSystems, Inc.	Cat#M768-tMEA-96OPT

### Resource availability

#### Lead contact

Further information and requests for resources and reagents should be directed to and will be fulfilled by the Lead Contact, Dr. Richard P. Davis (r.p.davis@lumc.nl).

#### Materials availability

Vectors for targeting the LP to AAVS1, as well as the base donor vectors and Cre recombinase expression vector have been deposited in Addgene. Catalog numbers are listed in the Key resources table. The hiPSC lines are available with an MTA.

### Experimental model and subject details

#### Ethics statement

Protocols for research involving human subjects and stem cell research were approved by the medical ethical committee at Leiden University Medical Center, the Netherlands.

#### hiPSC line culture

The hiPSC line LUMC0020iCTRL-06 (female, (Zhang et al., 2014), RRID: CVCL_ZA25) was generated from primary skin fibroblasts using Sendai virus by the LUMC hiPSC core facility. This line and the resulting subclones used in downstream experiments were assessed for pluripotency, tested for mycoplasma, DNA fingerprinted by STR analysis and karyotyped by G-banding. For each cell line, 20 metaphase spreads were examined with samples of sufficient quality to detect numerical and large structural abnormalities. For alkaline phosphatase staining, the alkaline phosphatase detection kit (Merck) was used following manufacturer’s instructions.

All hiPSC lines were maintained in StemFlex Medium (ThermoFisher) on laminin-521 (LN521; BioLamina)-coated (1.5 μg/cm^2^) plates. Cells were passaged twice a week by dissociating with either 1x TrypLE Select (ThermoFisher) or Accutase® solution (Sigma).

### Method details

#### hiPSC transfections

Intracellular delivery of DNA, RNA or protein into hiPSCs was accomplished by either electroporation or lipofection using conditions previously described (Brandão et al., 2021). Electroporation, (protocol #6 (1100 V, 30 ms, 1 pulse) of the Neon Transfection System (ThermoFisher)), was used to deliver Cas9-gRNA RNP complexes along with targeting constructs, or for delivering the BAC_attB(bxb) vector. All other transfections were performed using Lipofectamine™ Stem Transfection Reagent (ThermoFisher).

#### hiPSC subcloning

Dissociated hiPSCs were filtered to remove cell aggregates before being clonally isolated using the single-cell deposition function of a BD FACSAria™ III (BD Biosciences). Here, single hiPSCs were deposited directly into each well of an LN521-coated (1.8 μg/cm^2^) 96-well plate. To assist with clonal recovery, the culture media contained RevitaCell™ Supplement (1:100, ThermoFisher) for 72h as well as the anti-microbial Primocin® (InvivoGen) for 7 days. Media was changed every 3 days for 2 weeks, after which the cells were replicated for screening and archiving (Brandão et al., 2021).

#### Genomic DNA (gDNA) extraction

For hiPSCs cultured in 96 well-plates, gDNA was extracted using QuickExtract™ (Lucigen). Cells were resuspended in 30 μL QuickExtract and incubated at 65°C for 15 min, followed by 68°C for 15 min and 98°C for 10 min. For hiPSCs cultured in other formats, gDNA was extracted using the High Pure PCR Template Preparation Kit (Roche) and treated with RNAse A (10 mg/mL, Invitrogen) according to the manufacturers’ instructions.

#### Cas9 RNP & sgRNA synthesis

Cas9 protein was either purchased (IDT) or kindly provided by N. Geijsen (D’Astolfo et al., 2015). Candidate gRNAs with high specificity were identified around the intended mutation site using the bioinformatics tool, CRISPOR (Concordet and Haeussler, 2018), or were previously published (Wang et al., 2015). The gRNAs were synthesised as chimeric single gRNAs (sgRNAs) by *in vitro* transcription using the EnGen® sgRNA Synthesis Kit, *S. pyogenes* (NEB).

#### Golden gate (GG) reaction

Components amplified by PCR from DNA vectors were treated with DpnI (NEB) before purifying the PCR product using the Wizard® SV Gel and PCR Clean-Up System kit (Promega). All oligonucleotides were 5′ phosphorylated. Components that were used in multiple constructs (e.g. pCAG and pA signal) were also cloned into a pSMART vector backbone using the CloneSmart® HCKan Cloning Kit (Lucigen). The GG assembly reactions were performed in a total volume of 15 μL and included 80 fmol of each component, 40 fmol destination vector, 5 U restriction enzyme (ThermoFisher or NEB), 200 U T4 DNA Ligase (NEB) in ligation buffer with 100 μg/mL bovine serum albumin (BSA)(Bovogen Biologicals Australia). The reaction was performed in a thermocycler using the program: 37°C for 3 min then 16°C for 4 min (30 cycles); followed by steps of 37°C, 50 and 80°C, each for 5 min. To remove unligated DNA fragments, the reaction mix was treated with Plasmid-Safe™ ATP-Dependent DNase (Lucigen). Finally, 5 μL of the resulting reaction was transformed into competent *E. coli* strains as indicated below.

#### Bxb1-LP and φC31-LP cassette construction

The components of both cassettes were first PCR-amplified from other vectors and subsequently ligated together using the NEBuilder® HiFi DNA Assembly Cloning kit (NEB) to generate the resulting pENTR-eGFP-attP(bxb)-∗BsdR and pENTR-mCherry-attP(C31)-∗BleoR vectors (Addgene #183751 and #183752). Briefly, pENTR-eGFP-attP(bxb)-∗BsdR was composed of the backbone of the cloning vector pENTR/D-TOPO, a *loxP* sequence together with a PGK promoter, an *EGFP* reporter, and a blasticidin resistance gene lacking an initiation codon (∗*BsdR*). The primers included overlap sequences between adjacent fragments and were also used to introduce the *lox257* and Bxb1-specific *attP* sequences into the final vector. Similarly, pENTR-mCherry-attP(C31)-∗BleoR consisted of the same backbone vector, an *FRT* sequence together with a PGK promoter, a *mCherry* reporter, and a bleomycin resistance gene lacking an initiation codon (∗*BleoR*). Again, the primers included overlap sequences between adjacent fragments and were used to introduce the *F3* and φC31-specific *attP* sequences into the final vector.

#### Generation of *AAVS1* acceptor hiPSC lines

To target the Bxb1-LP and φC31-LP cassettes to the adeno-associated virus integration site (*AAVS1*) within intron 1 of *PPP1R12C*, the cassettes were PCR-amplified with primers containing overlap sequences to clone via NEBuilder® HiFi DNA Assembly into AAVS1_SA_2A_*Neo*_CAG_RTTA3 (Addgene, #60431) (Sim et al., 2014) digested with HindIII (NEB). The resulting targeting vectors AAVS1-Bxb1-LP-TC and AAVS1-φC31-LP-TC (Addgene #183754 and #183755), therefore, had the Bxb1-LP and φC31-LP cassettes flanked by ∼800 bp homology arms. Either AAVS1-Bxb1-LP-TC and/or AAVS1-φC31-LP-TC along with Cas9-*AAVS1* gRNA RNP complex (gRNA: 5′-GGGGCCACTAGGGACAGGAT-3′) were electroporated into LUMC0020iCTRL-06 hiPSCs. Following recovery and expansion of the electroporated cells, EGFP^+^, mCherry^+^ or double-positive hiPSCs were clonally isolated. Targeted clones were identified by PCR screening over the 5′ and-3′ homology arms.

#### Donor cloning vector construction

Three Bxb1 donor vectors were constructed for the cloning of various DNA payloads. For inserting DNA payloads <20 kb, the pBR_attB(bxb)_lox cloning vector was used (Figure S1C, *left*). This vector was built by digesting the plasmid, pEFBOS_creIRESBsd, with EcoRI (NEB) and ligating with a gBlock that contained a Bxb1-specific *attB* site, as well as *loxP* and *lox257* sequences. The cloning vector, p15_attB(bxb)_lox, was constructed for the cloning of 20–50 kb DNA payloads (Figure S1C, *middle*). A DNA sequence containing an ampicillin resistance cassette as well as the p15 origin of replication was PCR-amplified and inserted into the pBR_attB(bxb)_lox donor vector that had been linearized with PvuI and NsiI (both NEB) using NEBuilder HiFi DNA Assembly.

The cloning vector, pBR_attB(bxb)_ccdB_lox, was developed for modular construction of multi-component synthetic circuits (Figure S1C, *right*). In the first step, 3 DNA fragments (EF1a promoter plus Bxb1-*attB* and *loxP* sequences; β-lactamase (lacZ) cassette; *lox257* sequence) were PCR-amplified using primers that incorporated the recognition site for BpiI and specific 4 nucleotide (nt) overhangs to ensure the correct orientation and sequence of fragments in the final construct. In addition, the primers amplifying the lacZ cassette included the recognition site for Esp3I and a further two unique 4 nt overhangs. The pAGM4673 plasmid (Addgene #48014) (Weber et al., 2011) and the 3 PCR products were assembled as a GG reaction using BpiI (ThermoFisher), and transformed into 10-beta Competent *E. coli* (NEB). For the second step, a *ccdB* counterselectable marker was PCR-amplified with primers containing the recognition site for Esp3I and 4 nt overhangs to enable the replacement of the lacZ cassette by GG assembly, thereby generating the pBR_attB(bxb)_ccdB_lox donor vector (Addgene #183763).

The φC31 donor vector, pBR_attB(C31)_FRT (Addgene #183761), was constructed by digesting pEFBOS_creIRESBsd with EcoRI (NEB) and ligating with a gBlock that contained a φC31-specific *attB* site, as well as *FRT* and *F3* sequences (Figure S1D).

The insertion of DNA payloads between ∼2 and 50 kb into either the pBR_attB(bxb)_lox or p15_attB(bxb)_lox donor vectors (Addgene #183762 and #183764) was performed by subcloning fragments from a BAC carrying the human *KCNH2* gene (RP11-10L20) via recombineering (Figure S1E) (Fu et al., 2010).

To modify RP11-10L20 to enable targeted integration of the complete BAC construct (Bxb1-BAC donor) into the *AAVS1-*Bxb1 hiPSCs, recombineering was used to replace the *loxP* sequence in the BAC with a PCR-amplified DNA fragment that included the EF1a promoter, Bxb1-*attB* and a kanamycin-resistance cassette.

#### Donor vector integration into *AAVS1* acceptor hiPSCs

Unless stated otherwise, 1.2 μg of the donor vectors along with 0.8 μg of the corresponding integrase-expressing plasmids, pCAG-NLS-HA-Bxb1 (Addgene #51271) (Hermann et al., 2014) and pCAG-φC31 (Addgene #62658) (Ohtsuka et al., 2015), were transfected by lipofection into *AAVS1-*Bxb1, *AAVS1-*φC31 and *AAVS1-*Dual hiPSC lines. For comparing the integration efficiency of donor vectors of different sizes, 35.76 fmol of each vector was transfected.

To integrate the modified RP11-10L20 BAC construct into the *AAVS1-*Bxb1 hiPSCs, Bxb1-BAC donor was co-electroporated with pCAG-NLS-HA-Bxb1. For both approaches, ∼3 days after transfection the cells were harvested and passaged so that they were ∼5% confluent the following day. To enrich for integrated hiPSCs, either blasticidin S hydrochloride (2 μg/mL, Sigma) or zeocin selection reagent (15 μg/mL, ThermoFisher) were added to the culture medium for a period of 12 and 5 days, respectively. Donor vector integration was confirmed via a PCR screening strategy to detect the formation of the two new recombination sites, *attR* and *attL.*

#### Auxiliary sequence excision

hiPSCs were transfected by lipofection with either an Flp-expressing plasmid (1.6 μg, pCAG_FlpoIRESpuro (Kranz et al., 2010)), a Cre-expressing plasmid (1.6 μg, pEFBOS_CreIRESpuro, Addgene #183812, (Davis et al., 2008)) or StemMACS Cre Recombinase mRNA (200 ng, Miltenyi Biotec). For hiPSCs transfected with the plasmids, selection with puromycin (1 μg/mL, Sigma) was initiated 24 h post-transfection and maintained for 48 h. Genotyping PCR was used to confirm that the *loxP*- and *lox257*-or the *FRT*- and *F3*-flanked sequences were excised.

#### Droplet digital PCR (ddPCR)

ddPCR was performed and analyzed using a thermocycler, the Q200 AutoDG and QX200 Droplet Digital PCR System, and QuantaSoft software (all Bio-Rad). Assays comprising of premixtures of a forward and reverse primer (18 μM each) with a FAM- or HEX-conjugated hydrolysis probe (5 μM) were either purchased from Bio-Rad, based on previous publications (Roberts et al., 2017), or designed based on pre-defined criteria (Bell et al., 2018). Details regarding the assays are listed in Table S4. Reactions (final volume 22 μL) were prepared with 2x ddPCR Supermix for Probes (no dUTP, Bio-Rad), 900 nM of each primer, and 250 nM of each probe. To this, either 30–100 ng of gDNA digested with 2–5 U of HindIII, HaeIII or MseI (all NEB) depending on the sequence of the amplicon, or 3 μL of complementary DNA (cDNA) digested with MseI was added. Droplet generation, PCR amplification and analysis were all performed according to the manufacturer’s instructions. For CNV assays, the two-copy autosomal gene *RPP30* gene was used as a reference.

##### attP:attR assay

To determine the recombination efficiency of the donor plasmids into the Bxb1-LP or φC31-LP, probes were designed to detect the *attP* or *attR* sites in the transfected hiPSCs. For both events, the forward primer was common, while the reverse primers were specific for either the non-integrated or integrated locus. For amplicons amplified from the non-integrated population only one of the probes annealed, while for amplicons obtained from the integrated population both probes hybridized resulting in a stronger fluorescent signal that made it possible to distinguish droplets from each target. To optimise amplification conditions and confirm the specificity of the assay, gBlocks matching the two expected amplicons for each integrase were mixed in differing ratios and used as template DNA, with a strong correlation (R^2^ = >0.99) seen between the expected and observed frequencies (Figure S2).

##### KCNH2 variants

Probes were designed for each of the 12 *KCNH2* variants. To improve target specificity, 2–5 locked nucleic acids were included per probe. Amplification conditions for each probe were optimised for discriminating amplicons containing that specific missense mutation. Droplets were analyzed using the “absolute quantification” option of the QuantaSoft software.

##### KCNH2 allele-specific gene expression

To distinguish *KCNH2* transcripts from the wildtype allele and the allele reintroduced by STRAIGHT-IN, probes were developed to detect a heterozygous synonymous mutation present in exon 6. The FAM-conjugated probe was designed to recognise the *KCNH2* wildtype allele sequence (F513F), while the HEX-conjugated probe specifically binds to a silent mutation in F513F (C>T) only present in the reintroduced allele. RNA was extracted from hiPSC-CMs with the NucleoSpin RNA (Macherey Nagel) and DNA-*free*™ DNA Removal kits (ThermoFisher) according to manufacturers’ instructions, and transcribed into cDNA using the iScript™ cDNA Synthesis Kit (Bio-Rad).

#### Whole genome sequencing (WGS)

WGS was performed by GenomeScan (Leiden) with the library constructed using the NEBNext® Ultra II FS DNA and Ligation kits to fragment, A-tail and ligate sequencing adapters to the gDNA. The size of the resulting product was consistent with the expected size of approximately 500–700 bp. Clustering and DNA sequencing using the NovaSeq6000 platform (Illumina) was performed according to manufacturer’s protocols. A concentration of 1.1 nM of DNA was used. Image analysis, base calling, and quality check was performed with the Illumina data analysis pipeline RTA3.4.4 and Bcl2fastq v2.20.

#### Targeted capture sequencing

RNA capture probes were prepared by digesting the KCNH2 wildtype donor vector (p15-attB_KCNH2_wt_donor) with NlaIII to produce head to tail probes of an average length of 400 bp. The digested fragments were ligated with a Y-adapter containing the T7 sequence (T7_ada_top: 5-GGATTCTAATACGACTCACTATAGGGATGACCACCATCCGACT-3′, T7_ada_bot:/5Phos/GTCGGATGGTGGTCATAGCTGT-3) using the Kapa Hyper Prep kit (Roche) according to manufacturer’s instructions. The adapter ligated fragments were further amplified using the Kapa Hifi PCR mastermix (Roche) with the primers T7_adapter_for (5-GGATTCTAATACGACTCACTATAGGG-3′) and T7_adapter_rev (5-AGCGTGCAGGAAACAGCTATGACC-3′). The PCR reaction was purified using the Ampure Beads with a size selection of 0.8x. The sample was quantified using the Qubit 2.0 fluorometer (Thermo Fisher) and checked for size on a High Sensitivity DNA labonachip (Agilent) for adapter dimer content. To generate the biotinylated RNA probes, 50 ng of the purified PCR samples was transcribed using T7 RNA polymerase (NEB, M0251L), NTPs (ThermoFisher, R0481) and Bio-16-UTP (ThermoFisher). The Bio-16-UTP was mixed 50:50 with the regular TTP nucleotide. The reaction was incubated at 37°C for 16h. Prior to purifying the reaction, 1ul of RNAse free DNAse (Agilent) was added and incubated for 15 min at 37°C to remove the template DNA. The RNA was purified using the RNAClean XP beads (Beckman Coulter) and eluted in nuclease-free water before quantification using the Qubit 2.0 fluorometer and size assessment using the Nano Labonachip (Agilent).

For each cell line, 500 ng of gDNA was fragmented by sonication, followed by Illumina library prep using the KAPA Hyper Kit and unique barcoding of each sample with IDT adapters (xGen UDI-UMI adapters). The samples were purified twice to remove any adapter dimers and fragments below 150bp. The adapter ligated samples were further PCR enriched using the p5 and p7 primers for 10x cycles using the KAPA HiFi mastermix.

The Hyb Module box of the SureSelectQXT Target Enrichment for the Illumina Platform (Agilent) was used for hybridization and capturing of each sample separately. Briefly, 750 ng of the Illumina-prepped sample and 500 ng of the biotinylated RNA capture probes were used in the reaction. A 15 cycle post capture PCR with the p5 and p7 primers was performed using the KAPA HiFi mastermix, with the resulting samples purified using 0.8x Ampure XP beads (Beckman Coulthier) and quantified using the Qubit 2.0 (ThermoFisher) followed by size assessment using the High Sensitivity DNA labonachip (Agilent). The samples were pooled in equimolar ratios and sequenced on the NovaSeq 6000 using the 1.5v reagent kit (2 × 150bp) following the vendor’s instructions.

#### Sequencing data processing

Both WGS and amplicon sequencing data were processed using the BioWDL germline DNA pipeline developed at LUMC (https://github.com/biowdl/germline-DNA). This pipeline includes quality control by FastQC (v0.11.9), adapter clipping using Cutadapt (v2.8), alignment to the human reference genome GRCh38 using BWA-MEM (v0.7.17), removal of UMI-based duplicate reads using Picard (v2.23.2) and variant detection using GATK4 best practice workflow (v4.1.8.0). Copy number analysis was performed using Control-FREEC (v11.6). Two regions of interest were defined based on examining the WGS coverage tracks using IGV: a copy number normal region consisting of chr7:150848406-150936295 and chr7:150986192-151010515; and a copy number loss region of chr7:150936295-150986192. To calculate variant allele frequency (VAF) in these two regions, a customized Python script was developed using CyVCF2 to check all SNPs' variant frequency by examining the SNP sites. Using the Python package of matplotlib, violin plots are created to compare VAF of different samples and regions.

#### Optogenetic reporter hiPSC line generation

The assembly of the individual and multi-parameter reporter donor vectors was based on the modular and hierarchal cloning system, MoClo (Weber et al., 2011). Briefly, the required components for the expression of these reporters (i.e. promoter sequence, localisation signal, CDS and polyA signal) were PCR-amplified using primers that incorporated the recognition site for the type IIS enzyme BsaI and previously designated overhang sequences for positioning and orientation of the components (Andreou and Nakayama, 2018; Weber et al., 2011). The only exception was the CDS for jRCaMP1b, which was synthetically designed to introduce silent mutations to destroy BpiI and BsaI recognition sites present in the original CDS.

The components for each of the individual optogenetic sensors were first assembled in Level 1 destination vectors included in the MoClo Toolkit (Addgene #1000000044) by GG assembly with the restriction enzyme, FastDigest Eco31I (ThermoFisher), thereby generating the intermediate Transcriptional Unit (TU) vectors, which were transformed into 10-beta Competent *E. coli* (NEB). The reporter construct for ASAP2f was assembled into 2 different Level 1 destination vectors, while the constructs for jRCaMP1b and miRFP703 were each assembled into Level 1 destination vectors for positions 2 and 3 respectively.

The resulting TU vectors were subsequently assembled either individually or in combination into the donor vector pBR_attB(bxb)_ccdB_lox by digestion with BpiI. Dummy and end-linker vectors from the MoClo Toolkit were included as required in the GG assembly reaction. The ensuing donor vectors (Bxb1-ASAP2f, Addgene #183757; Bxb1-jRCaMP1b, Addgene #183758; Bxb1-miRFP703, Addgene #183759 and Bxb1-AJMA, Addgene #183760) were transformed into Stbl2™ *E. coli* (ThermoFisher) for ccdB counterselection.

Each donor vector was separately integrated into the *AAVS1-*Bxb1 hiPSCs, followed by excision of the auxiliary sequences. Clonal hiPSC lines were derived for the single reporter targeted cells, while enrichment for the *AAVS1*-AJMA hiPSCs was performed by flow cytometric sorting of cells co-expressing the 3 reporters. Genotyping PCRs confirmed targeted integration, as well as no rearrangement within the TUs of the *AAVS1*-AJMA hiPSCs.

#### *KCNH2*^+/^^A^^cc^ hiPSC line generation

The vector to target the Bxb1-LP cassette to the *KCNH2* locus (*KCNH2*-Bxb1-LP-TC) was generated by PCR-amplifying the Bxb1-LP cassette with primers containing 80 bp overhangs complementary to endogenous sequences ∼8.5 kb (5′) and ∼8.7 kb (3′) of *KCNH2*. The resulting PCR product was cloned into pMini T2.0 using the PCR Cloning Kit (NEB).

One copy of *KCNH2* was deleted by electroporating Cas9 protein together with two gRNAs targeting both ends (gRNA for 5′ end: 5′-ATGAAGGCTTTCCCATCCGT-3′ and gRNA for 3′ end: 5′-ACTGTGCTGGGTACGCTGAC-3′) into LUMC0020iCTRL-06. This was confirmed for a hiPSC clone by PCR screening and Sanger sequencing, with ddPCR CNV assays verifying that the clone was monoallelic for *KCNH2*. Next, the *KCNH2*-Bxb1-LP-TC along with a Cas9-*KCNH2* gRNA RNP complex (gRNA: 5′-CTGGTTGTGCTGACTGTGCT-3′) was electroporated into this modified hiPSC line. Following recovery and expansion of the electroporated cells, EGFP^+^ hiPSCs were clonally isolated. Targeted clones (*KCNH2*^+/Acc^) were further characterised by PCR screening and Sanger sequencing over the 5′ and -3′ homology arms. The resulting *KCNH2*^+/Acc^ hiPSC line selected contained a single integration event of the Bxb1-LP cassette as determined by ddPCR.

#### *KCNH2* donor vector construction

The donor vectors containing the various *KCNH2* genomic sequences were built based on recombineering strategies previously described (Fu et al., 2010; Wang et al., 2014). Briefly, to seamlessly introduce the variants into *KCNH2*, a counterselection cassette (ccdB-Amp) was first introduced to replace exon 7 in the BAC, RP11-10L20. Next, synthetic double-stranded DNA fragments that introduced specific missense mutations in exon 7 of *KCNH2* (Table S1) were pooled and amplified by PCR, before being electroporated into the bacteria to replace the counterselection cassette. Colonies that subsequently grew in the absence of L-arabinose (Sigma) were then screened by PCR and Sanger sequencing to identify recombined BACs for each of the 12 mutations.

The sequence in the BAC corresponding to the *KCNH2* genomic region deleted in the *KCNH2*^+/Acc^ hiPSCs was subsequently subcloned from both wildtype RP11-10L20 as well as clones carrying the introduced variants into p15_attB(bxb)_lox by recombineering. The resulting colonies were screened by PCR to confirm subcloning and the plasmids retransformed into Stable Competent *E. coli* (NEB) to ensure the resulting *KCNH2* donor vectors were pure. Sanger sequencing also confirmed the presence of each missense mutation, and that the *attB* and *lox* sequences were correct. Finally, the integrity of each of the donor vectors was evaluated by restriction analysis.

Bacterial cultures of the *KCNH2* variant donor vectors were pooled and grown as a 200 mL culture overnight at 30°C, while the KCNH2 wildtype donor vector (p15-attB_*KCNH2*_wt_donor) was cultured separately. Plasmid DNA was purified using the NucleoBond® Xtra Midi Kit (Macherey Nagel) following manufacturer’s instructions.

#### *KCNH2*-variant and WT hiPSC line generation

To generate the *KCNH2*^+/Acc-^^WT^ line, a similar procedure to that used to generate the *AAVS1*-reporter hiPSCs was performed, except that 3.2 μg of p15-attB_*KCNH2*_wt_donor was transfected into the *KCNH2*^+/Acc^ hiPSCs. Cells were selected with blasticidin for 6 days, followed by transfection of pEFBOS_CreIRESpuro and selection with puromycin. The resulting *KCNH2*^+/Acc-^^WT^ hiPSCs were clonally isolated by single-cell deposition and identified by genotyping PCR.

To multiplex the generation of the *KCNH2*^+/^^A^^cc-variant^ lines, 0.6 μg of the pooled 12 *KCNH2* variant donor vectors was transfected because of toxicity observed when transfecting higher amounts of plasmid DNA. The antibiotic selection strategy was also modified, with the cells maintained in culture medium containing 2 μg/mL blasticidin over 3 passages (14 days). Excision of the auxiliary sequences was performed by Cre recombinase mRNA transfection. Following single-cell deposition, genotyping PCR detected clones that had undergone both integration and excision steps, while Sanger sequencing identified the heterozygous *KCNH2* variant that was introduced.

#### Differentiation and culture of hiPSC-CMs

The hiPSCs were differentiated into cardiomyocytes as previously described (Campostrini et al., 2021). One day prior to differentiation (d-1), the hiPSCs were harvested using TrypLE Select and plated onto Matrigel (1:100, Corning)-coated wells in StemFlex™ Medium containing RevitaCell™ Supplement (1:200 dilution). On d0, the cells were refreshed with mBEL medium containing 5 μM CHIR99021 (Axon Medchem). On differentiation d2, the cells were refreshed with mBEL medium containing 5 μM of XAV939 (Tocris) and 0.25 μM IWP-L6 (AbMole). From differentiation d4 on, the cells were maintained in mBEL medium. The hiPSC-CMs were cryopreserved at differentiation d20 or d21 as previously described in a freezing medium comprising of 90% Knockout Serum Replacement (Gibco) and 10% DMSO (Brink et al., 2020). Subsequent thawing and seeding of the cells were performed as previously described (Brink et al., 2020; Campostrini et al., 2021).

#### Flow cytometric analysis

A single-cell suspension of hiPSCs or hiPSC-CMs was obtained by dissociating the cells with 5x TrypLE Select and filtering the cell suspension. Cells were fixed and permeabilised using the FIX and PERM™ Cell Permeabilization Kit (ThermoFisher) according to manufacturer’s instructions. The hiPSC-CMs were incubated with the conjugated antibodies cTnT-Vioblue or cTnT-FITC (1:50, Miltenyi Biotec, #130-120-402 or #130-119-575). All antibodies were diluted in permeabilization medium (medium B; ThermoFisher). The data was acquired using a MacsQuant VYB flow cytometer (Miltenyi Biotec) and analyzed using FlowJo software (v. 10.2, FlowJo).

#### Fluorescence imaging

The hiPSCs and hiPSC-CMs were seeded on 96-well imaging microplates (Corning), with images of the fluorescent reporters acquired using an EVOS™ M7000 Cell Imaging System (ThermoFisher) at 40× magnification. For visualizing the sarcomeres, the hiPSC-CMs were fixed and permeabilised with the FIX and PERM™ Cell Permeabilization kit and labelled with an antibody specific for α-actinin (1:250, Sigma-Aldrich #A7811), followed by an Alexa Fluor 350-conjugated secondary antibody (1:500, ThermoFisher, #A-11045).

#### Optical evaluation of hiPSC-CMs

4–5 × 10^4^ hiPSC-CMs differentiated from the optogenetic reporter hiPSCs were seeded per well on 96-well imaging microplates pre-coated with Matrigel (1:100) in mBEL medium. Medium was refreshed the next day and every 2–3 days thereafter, with analysis performed 7 days after thawing. Before performing baseline measurements, wells were refreshed with 200 μL mBEL medium and left for 60 min at 37°C to equilibrate.

After baseline measurements, the hiPSC-CMs were refreshed with 100 μL of mBEL including compounds at final test concentrations and incubated for 5 min at 37°C before recording. Compounds used were E-4031, verapamil (both Tocris Bioscience) and isoprenaline (Sigma-Aldrich). All compounds were reconstituted in DMSO (Sigma-Aldrich), with solutions prepared to ensure a final concentration of 0.1% DMSO in each well. Vehicle incubations were done similarly using mBEL +0.1% DMSO. Measurements were made with cells paced at 1.2 Hz using a pair of field stimulation electrodes placed in the culture medium, except for the isoprenaline measurements which were performed on spontaneously beating hiPSC-CMs. A Leica DMI6000B imaging system (Leica Microsystems) equipped with 470, 565 and 656 nm lasers was used to record signals for AP, cytosolic Ca^2+^ and contraction transients respectively, at 40 frames per second. The microscope was fitted with an environmental chamber that allowed for the measurements to be performed at 37°C and 5% CO_2_. The analyses for AP and cytosolic Ca^2+^ transients were performed using ImageJ (NIH) and algorithms developed in-house (Meer et al., 2019). For contraction transients, the analyses were performed using the software, MUSCLEMOTION (Sala et al., 2018).

#### hiPSC-CM multielectrode array (MEA) recordings

The hiPSC-CMs were seeded (4–6 × 10^4^ cells/well) in a Matrigel-coated 96 well Lumos MEA plate (Axion BioSystems, Inc.) in 5 μL mBEL medium supplemented with RevitaCell™ (1:200). The cells were incubated for 1 h at 37°C, 5% CO_2_ to allow attachment, with the wells then supplemented with an additional 150 μL mBEL medium. The medium was refreshed the next day and every 2–3 days thereafter.

Recordings were performed using the Maestro Pro multiwell MEA platform (Axion BioSystems, Inc.). Field potential (FP) recordings were either performed on spontaneously beating hiPSC-CMs (*KCNH2*^*+/+*^ vs *KCNH2*^*+/Acc-*^^*WT*^) or on optically paced cells (*KCNH2*^*+/Acc-*^^*WT*^ vs *KCNH2*^*+/Acc-A561T*^). Optical pacing of the hiPSC-CMs was performed as previously described (Brink et al., 2021). Briefly, at day 8 or 9 post-seeding the hiPSC-CMs were transfected with 50 ng of *in vitro* transcribed mRNA (Yiangou et al., 2022) encoding Channelrhodopsin-2 (ChR2) using Lipofectamine Stem Transfection Reagent. Medium was refreshed ∼18 h post-transfection. 2 or 3 days post-transfection, and at least 1 h before recordings, medium was refreshed with mBEL supplemented with 1 μM all-*trans* retinal (Sigma). Subsequently, the hiPSC-CMs were paced at 1.25 Hz using 10 ms pulses of blue light (475 nm) delivered for 5 min using the Lumos™ Optical Stimulation System (Axion Biosystems, Inc.).

Prior to all recordings, the hiPSC-CMs were equilibrated inside the device at 37°C, 5% CO_2_ for 10 min. Recordings were performed for 3–4 min using Cardiac Standard filters and amplifiers in spontaneous cardiac mode (12.5 kHz sampling frequency; 0.1–2000 Hz band-pass filter). Raw data files were re-recorded to generate CSV files using the AxIS digital filters (Butterworth: 0.1 Hz (high) and 2 kHz (low)) and the following cardiac beat detector settings: amplitude threshold: 80–150 μV; inflection search (detection Auto (Max/Min), hold off: 50 ms (post), 100 ms (pre), max post-search duration: 1s); and statistic compiler (30 stable beats selection, no FPD quality control). CSV and RAW files were loaded into the Cardiac Analysis Tool (Axion BioSystems, Inc., version 3.1.5) to enable precise assessment and analysis of the FP duration (FPD) from one “golden electrode” per well.

### Quantification and statistical analysis

Data is presented as mean ± SEM unless otherwise noted. Statistical analysis was performed using GraphPad Prism 8 software (v8.2.0, GraphPad). Sample sizes, statistical analyses and p values are reported in the figure legends. Differences were considered statistically significant at p < 0.05.

## Data Availability

• DNA sequencing datasets have been deposited in the ArrayExpress database at EMBL-EBI (https://www.ebi.ac.uk/biostudies/arrayexpress/) under accession number E-MTAB-11971. • This paper does not report original code. • Any additional information required to reanalyze the data reported in this paper is available from the lead contact upon request.

## Supplemental information

Document S1. Figures S1–S6 and Tables S1–S4

Document S2. Article plus supplemental information
