# Supplementary material for: STRAIGHT-IN enables high-throughput targeting of large DNA payloads in human pluripotent stem cells
Source: Cell Rep Methods. 2022 Sep 22;2(10):100300. doi: 10.1016/j.crmeth.2022.100300 (PMC9606106; doi:10.1016/j.crmeth.2022.100300)
Supplement: Document S1. Figures S1–S6 and Tables S1–S4 [file mmc1.pdf]

Cell Reports Methods, Volume *2*

Supplemental information

STRAIGHT-IN enables high-throughput targeting of large DNA payloads in human pluripotent stem cells

Albert Blanch-Asensio, Catarina Grandela, Karina O. Brandão, Tessa de Korte, Hailiang Mei, Yavuz Ariyurek, Loukia Yiangou, Mervyn P.H. Mol, Berend J. van Meer, Susan L. Kloet, Christine L. Mummery, and Richard P. Davis

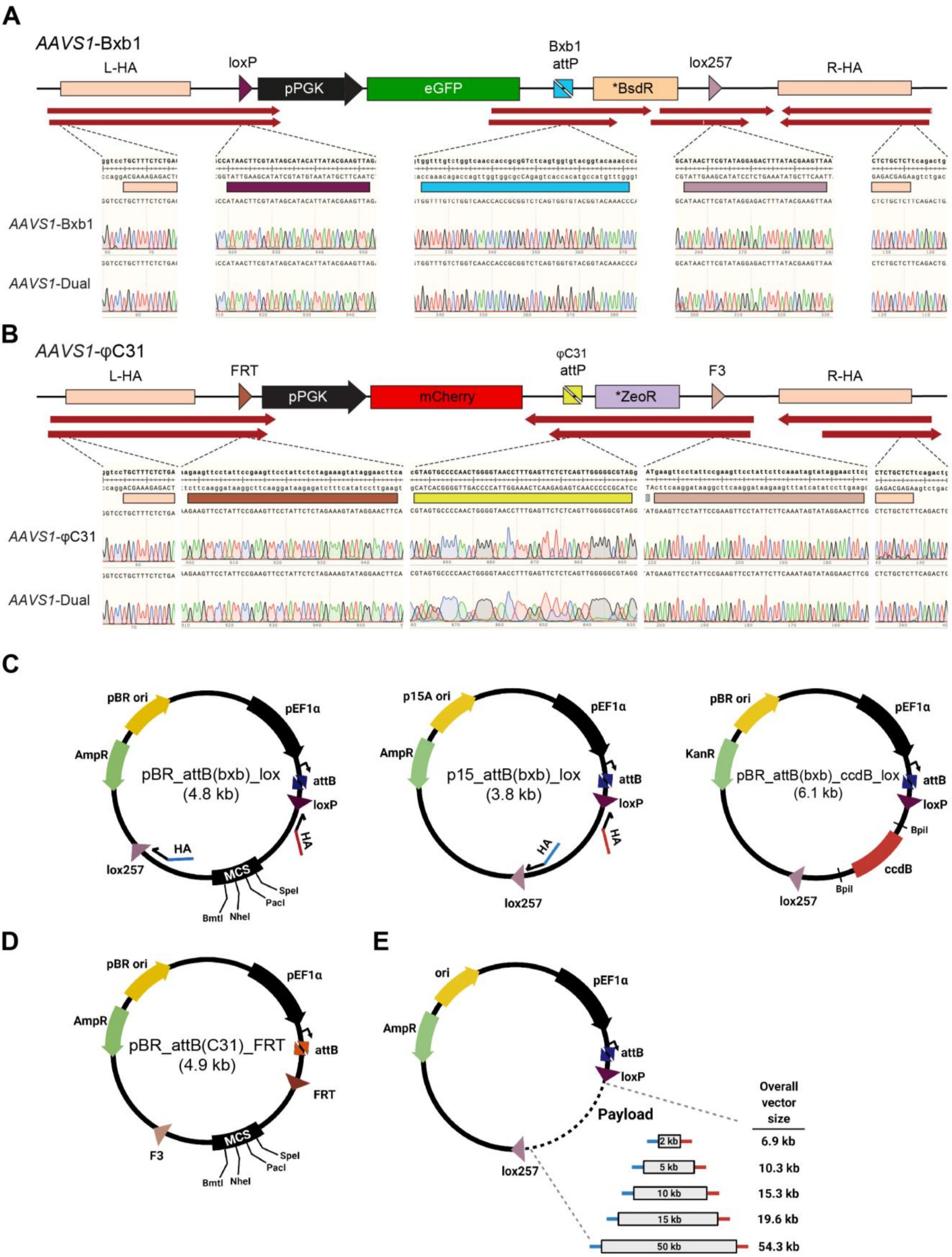
A
AAVS1-Bxb1
L-HA
loxP
pPGK
eGFP
Bxb1
attP
*BsdR
lox257
R-HA
AAVS1-Bxb1
AAVS1-Dual
B
AAVS1-φC31
L-HA
FRT
pPGK
mCherry
φC31
attP
*ZeoR
F3
R-HA
AAVS1-φC31
AAVS1-Dual
C
pBR ori
pEF1α
AmpR
pBR_attB(bxb)_lox
(4.8 kb)
attB
loxP
HA
lox257
HA
MCS
SpeI
PacI
BmtI
NheI
p15A ori
pEF1α
AmpR
p15_attB(bxb)_lox
(3.8 kb)
attB
loxP
HA
HA
lox257
pBR ori
pEF1α
KanR
pBR_attB(bxb)_ccdB_lox
(6.1 kb)
attB
loxP
BpiI
ccdB
BpiI
lox257
D
pBR ori
pEF1α
AmpR
pBR_attB(C31)_FRT
(4.9 kb)
attB
FRT
F3
MCS
SpeI
PacI
BmtI
NheI
E
ori
pEF1α
AmpR
attB
loxP
Payload
lox257
Overall vector size
2 kb
6.9 kb
5 kb
10.3 kb
10 kb
15.3 kb
15 kb
19.6 kb
50 kb
54.3 kb

Figure S1. Overview of the hiPSC *AAVS1*-acceptor lines and the donor vectors. Related to Figure 1 and STAR Methods.

(A) Sanger sequencing confirming targeting of the Bxb1-LP to the *AAVS1* locus and the sequences of the *loxP*, *lox257* and *attP* (Bxb1) sites in the *AAVS1*-Bxb1 and *AAVS1*-Dual hiPSC lines. Red arrows indicate alignment of the sequencing chromatograms. L-HA, left homology arm; R-HA, right homology arm.

(B) Sanger sequencing confirming targeting of the φC31-LP to the *AAVS1* locus and the sequences of the *FRT*, *F3* and *attP* (φC31) sites in the *AAVS1*-φC31 and *AAVS1*-Dual hiPSC lines. Red arrows indicate alignment of the sequencing chromatograms. L-HA, left homology arm; R-HA, right homology arm.

(C) Schematics of the Bxb1 donor vectors used for inserting the DNA payloads and their respective sizes (kilobases, kb). The pBR_attB(bxb)_lox plasmid (*left*) was used for cloning payloads <20 kb, either by enzymatic digestion and ligation using restriction enzymes indicated within the multi-cloning site (MCS), or by recombineering. The p15_attB(bxb)_lox plasmid (*middle*) was used for cloning payloads between ~20-50 kb by recombineering. The pBR_attB(bxb)_ccdB_lox plasmid (*right*) was used for cloning payloads via modular assembly strategies involving digestion of the plasmid with the type IIS restriction enzyme, BpiI. Half arrows, recombineering primers used to amplify the cloning vector with homology arms (HA) to the DNA payload attached; pEF1a; human elongation factor 1 alpha promoter; ori, origin of replication; AmpR, b-lactamase; KanR, aminoglycoside phosphotransferase.

(D) Schematic of the φC31 donor vector (pBR_attB(C31)_FRT). pEF1a; human elongation factor 1 alpha promoter; ori, origin of replication; AmpR, b-lactamase.

(E) Overview of the Bxb1-donor vectors containing DNA payloads between ~2 – 50 kb, and the resulting size of the vector.

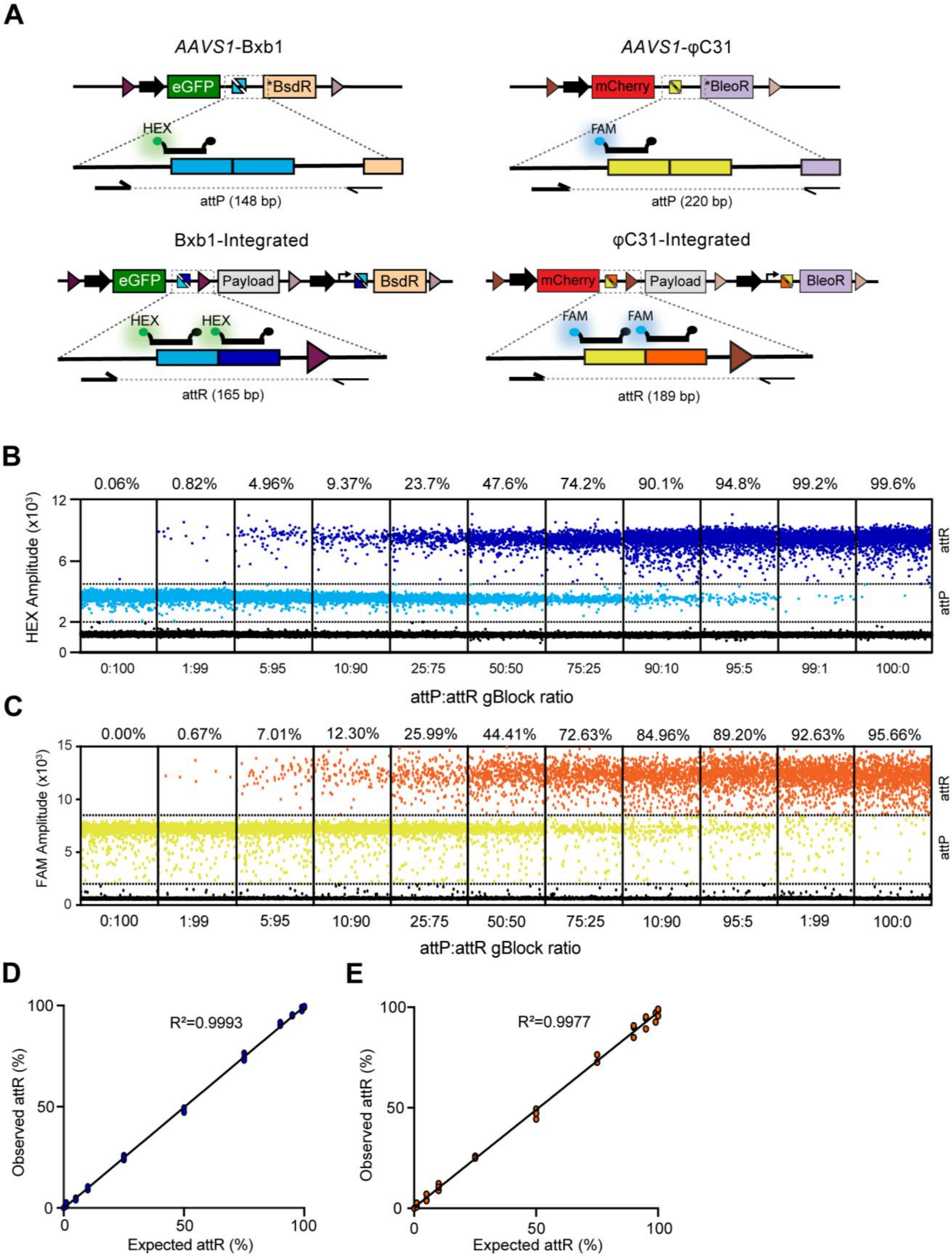
A
AAVS1-Bxb1
eGFP
*BsdR
HEX
attP (148 bp)
AAVS1-φC31
mCherry
*BleoR
FAM
attP (220 bp)
Bxb1-Integrated
eGFP
Payload
BsdR
HEX
HEX
attR (165 bp)
φC31-Integrated
mCherry
Payload
BleoR
FAM
FAM
attR (189 bp)
B
0.06% 0.82% 4.96% 9.37% 23.7% 47.6% 74.2% 90.1% 94.8% 99.2% 99.6%
HEX Amplitude (x10³)
attR
attP
0:100 1:99 5:95 10:90 25:75 50:50 75:25 90:10 95:5 99:1 100:0
attP:attR gBlock ratio
C
0.00% 0.67% 7.01% 12.30% 25.99% 44.41% 72.63% 84.96% 89.20% 92.63% 95.66%
FAM Amplitude (x10³)
attR
attP
0:100 1:99 5:95 10:90 25:75 50:50 75:25 10:90 95:5 1:99 100:0
attP:attR gBlock ratio
D
R²=0.9993
Observed attR (%)
Expected attR (%)
E
R²=0.9977
Observed attR (%)
Expected attR (%)

Figure S2. Validation of *attP:attR* ddPCR assay for quantifying Bxb1- or φC31-mediated integration. Related to Figure 2 and STAR Methods.

(A) Schematic of the DNA regions amplified from non-integrated (*attP*, *upper*) and integrated cells (*attR*, *lower*) in the ddPCR assay for either Bxb1 (*left*) or φC31 (*right*) mediated integration. A common forward primer (thick half arrow) and sequence-specific reverse primers (thin half arrows) were used to amplify the PCR products. Either one or both fluorescence probes (thick black bar) could anneal to the two resulting amplicons, leading to differences in signal intensity.

(B, C) Representative ddPCR dot plots of the observed frequencies at different ratios for two synthetic sequences matching the expected *attP* and *attR* amplicons for either Bxb1 (B) or φC31 (C). Sequences were spiked into genomic DNA. Dots represent droplets containing the indicated sequence, while percentages denote the calculated integration efficiency.

(D, E) Regression analysis for the observed versus expected frequency of the Bxb1 (D) and φC31 (E) *attR* amplicons; n=3 replicates.

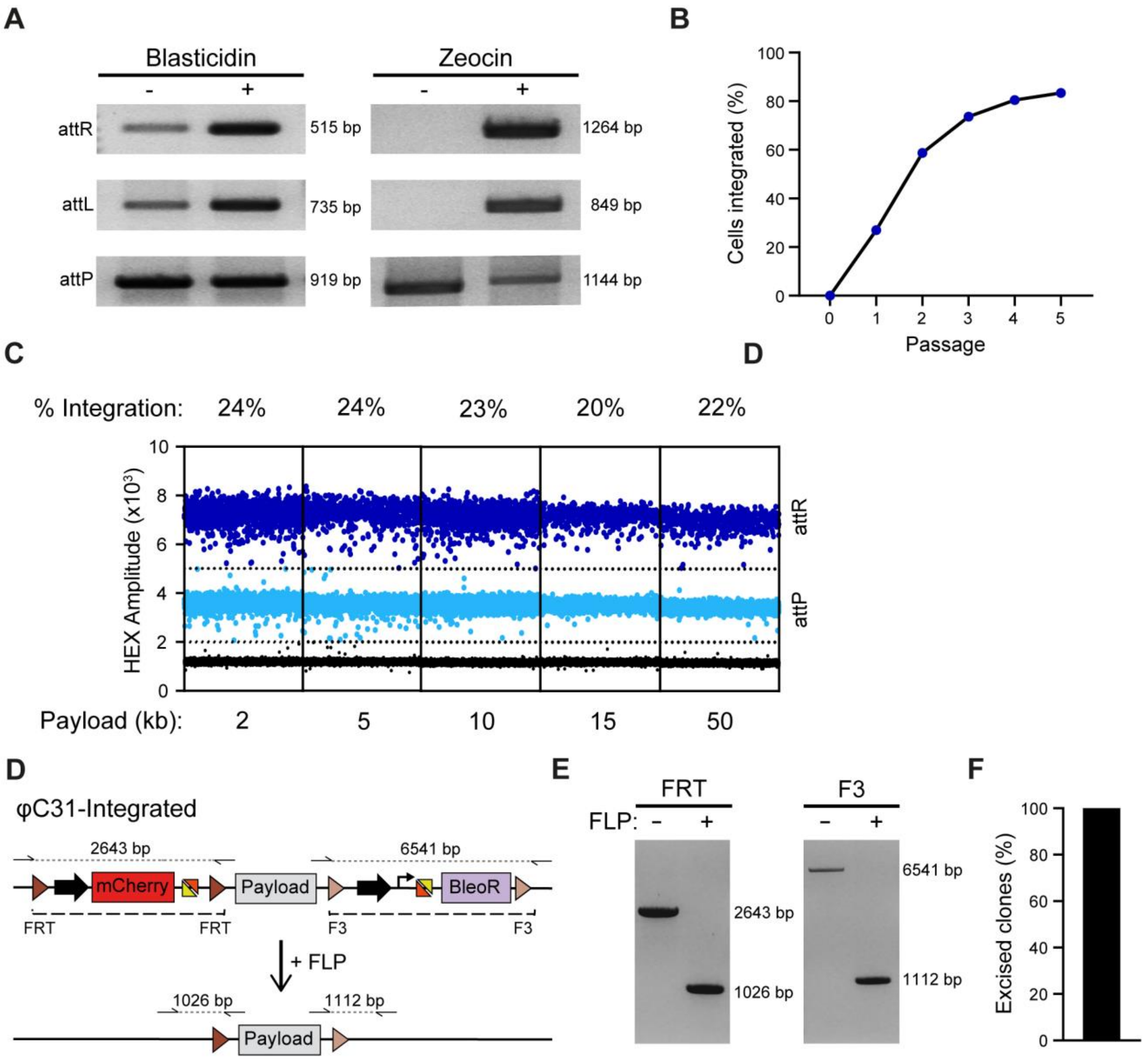

Figure S3. Evaluating the efficiency of integration and excision using STRAIGHT-IN. Related to Figures 2 and 4.

(A) PCR amplification of genomic DNA confirming integration of the Bxb1- and φC31-donor vectors into the *AAVS1*-Dual hiPSCs. The "-" and "+" symbols indicate before and after the corresponding antibiotic selection respectively.

(B) Percentage of cells with the Bxb1 donor vector integrated when blasticidin selection is maintained for 5 passages of the hiPSCs.

(C) Representative ddPCR dot plot of the percentage of hiPSCs that integrated donor constructs with payloads ranging from 2 – 50 kb into the Bxb1-LP following one round of blasticidin selection. The amount of the DNA delivered into the cells was adjusted based on the size of the plasmid.

(D) Schematic of procedure for excising the positive selection cassettes and vector backbone following integration of the donor vector into the φC31-LP. Dashed lines indicate the sequences excised. Half arrows indicate primer binding sites with dotted lines representing the resulting PCR amplicons.
(E) PCR screening using primer pairs indicated in (D), confirming the reduction in amplicon length upon expression of FLP recombinase (+).
(F) Quantification of integrated hiPSCs that have excised the auxiliary sequences following FLP expression.

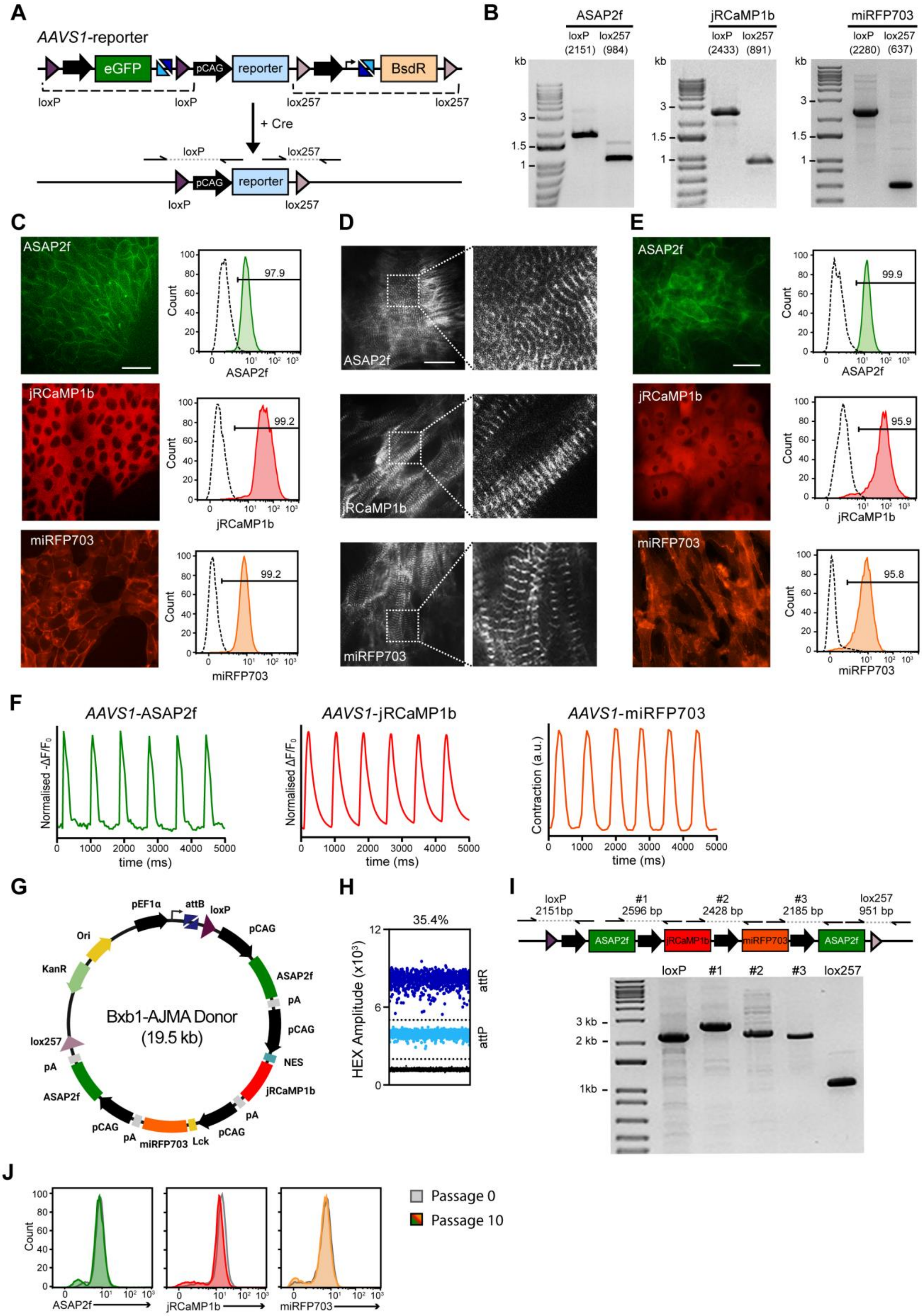

A
AAVS1-reporter
eGFP
pCAG
reporter
BsdR
loxP
lox257
+ Cre
B
ASAP2f
jRCaMP1b
miRFP703
loxP (2151)
lox257 (984)
loxP (2433)
lox257 (891)
loxP (2280)
lox257 (637)
kb
C
D
E
Count
97.9
99.2
99.2
99.9
95.9
95.8
F
AAVS1-ASAP2f
AAVS1-jRCaMP1b
AAVS1-miRFP703
Normalised -ΔF/F0
Normalised ΔF/F0
Contraction (a.u.)
time (ms)
G
Bxb1-AJMA Donor
(19.5 kb)
pEF1α
attB
Ori
KanR
pCAG
pA
NES
Lck
H
35.4%
HEX Amplitude (x10³)
attR
attP
I
loxP 2151bp
#1 2596 bp
#2 2428 bp
#3 2185 bp
lox257 951 bp
3 kb
2 kb
1kb
J
Passage 0
Passage 10

Figure S4. Assessment of genetic reporters introduced by STRAIGHT-IN for evaluating APs, Ca^{2+} transients and contractility kinetics. Related to Figure 5.

(A) Schematic of the composition of the *AAVS1* locus following the integration of the fluorescent reporters (*ASAP2f, jRCaMP1b, miRFP703*). Filled black arrows, constitutive promoters; eGFP, enhanced green fluorescence protein; BsdR, blasticidin resistance gene; dashed lines, sequences excised; half arrows, primer binding sites; dotted lines, resulting PCR amplicons generated by screening across the remaining *loxP* and *lox257* sites.

(B) PCR screening across regions indicated in (A) confirming targeted integration of each of the fluorescent reporters into the *AAVS1*-Bxb1 hiPSC line. The base-pair size of the expected amplicons for each reporter is indicated in brackets. A DNA ladder was loaded in the first column of each gel, with the size of selected bands indicated. kb, kilobase.

(C) Fluorescence images (*left*) and flow cytometric analysis (*right*) from the *AAVS1*-reporter hiPSC lines indicating the cellular localisation and expression of each of the integrated reporters. Scale bar, 50 µm.

(D) Immunofluorescence images of the cardiac sarcomeric protein α-actinin from each of the *AAVS1*-reporter hiPSC lines following differentiation to cardiomyocytes. Images on the right are magnifications of the regions within the dotted boxes. Scale bar, 25 µm.

(E) Fluorescence images (*left*) and flow cytometric analysis (*right*) of cardiomyocytes differentiated from the *AAVS1*-reporter hiPSC lines indicating the cellular localisation and expression of each of the integrated reporters. Scale bar, 50 µm.

(F) Representative time plots of baseline-normalised fluorescence signals from the *AAVS1*-reporter hiPSC-CMs stimulated at 1.2 Hz. Changes in the fluorescence of *AAVS1*-ASAP2f (*left*) and *AAVS1*-jRCaMP1b (*middle*) hiPSC-CMs reflect the action potential and cytosolic Ca^{2+} transients respectively, while the displacement of the fluorescence signal in *AAVS1*-miRFP703 hiPSC-CMs indicates contraction dynamics.

(G) Schematic of the donor vector for integrating two *ASAP2f* expression cassettes and single expression cassettes for *jRCaMP1b* and *miRFP703*. pEF1a; human elongation factor 1 alpha promoter; pCAG, CAG promoter; pA, polyadenylation signal; NES, nuclear export signal; Lck, Lck membrane targeting signal; ori, origin of replication; KanR, aminoglycoside phosphotransferase.

(H) ddPCR dot plot of *AAVS1*-Bxb1 hiPSCs transfected with the Bxb1-AJMA donor vector. Dots represent droplets containing the indicated sequence (attR or attP), while the percentage denotes the calculated integration efficiency.

(I) Schematic of the resulting *AAVS1*-AJMA locus (*top*). Half arrows, primer binding sites; dotted lines, resulting PCR amplicons with expected sizes indicated in brackets. PCR screening (*bottom*), using the primer pairs indicated, confirming targeted integration of the multi-reporter construct and no internal rearrangement of the transgenes. A DNA ladder was loaded in the first column of the gel, with the size of selected bands indicated. kb, kilobase.

(J) Flow cytometric analysis of the expression of the indicated reporters in the *AAVS1*-AJMA hiPSC line following 10 passages in culture.

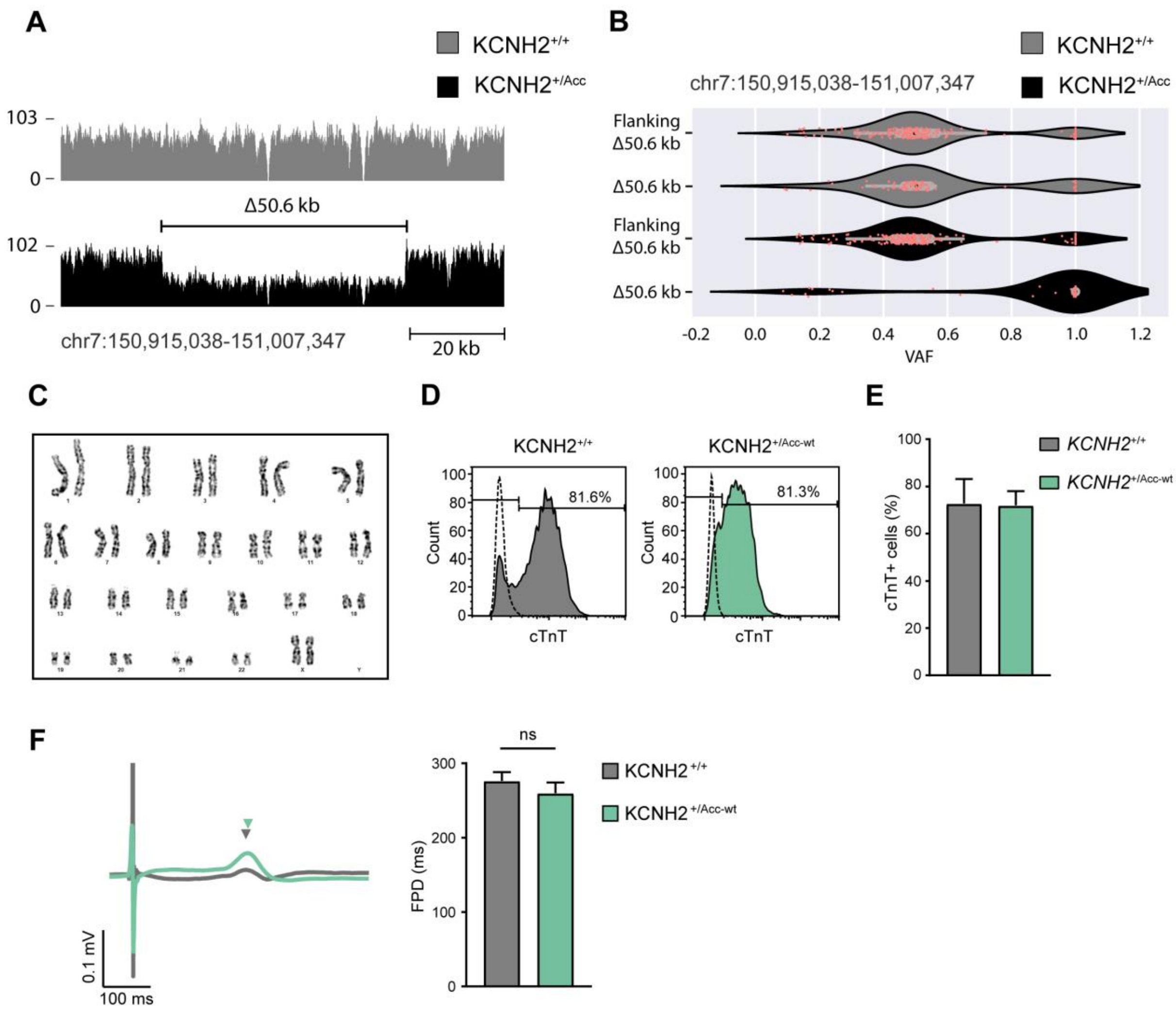

Figure S5. Characterising the *KCNH2*$^{+/Acc\text{-}wt}$ hiPSCs and hiPSC-CMs. Related to Figure 6.

(A) WGS of *KCNH2*$^{+/Acc}$ showed a reduced number of sequence reads over a 50.6 kb region on chromosome 7 that includes KCNH2, indicating deletion in one of the alleles.

(B) Analysis of variant frequencies confirmed the absence of heterozygous variants (VAF=0.5) only within the replaced 50.6 kb region in *KCNH2*$^{+/Acc}$ and not in the genome flanking this region or in *KCNH2*$^{+/+}$ hiPSCs.

(C) G-banding karyogram for a *KCNH2*$^{+/Acc\text{-}wt}$ hiPSC line generated by STRAIGHT-IN indicating a normal 46, XX karyotype.

(D) Representative histogram plots showing the percentage of cardiomyocytes (cTnT^{+}) as determined by flow cytometry following differentiation of either *KCNH2*$^{+/+}$ or *KCNH2*$^{+/Acc\text{-}wt}$ hiPSCs. Dotted lines represent a control cTnT^{-} population.

(E) Averaged percentage of differentiated *KCNH2*$^{+/+}$ and *KCNH2*$^{+/Acc\text{-}wt}$ hiPSCs that stained positive for cTnT. n=3 differentiations and error bars represent ± SEM.

(F) Representative averaged field potential (FP) traces (*left*) and averaged FP duration (FPD) values (*right*) of *KCNH2*$^{+/+}$ and *KCNH2*$^{+/Acc\text{-}wt}$ hiPSC-CMs. Arrowheads indicate the repolarization peak for each trace. n = 34 (*KCNH2*$^{+/+}$) and 25 (*KCNH2*$^{+/Acc\text{-}wt}$) recordings; error bars ± SEM; ns, not significant ($p > 0.05$; unpaired t-test).

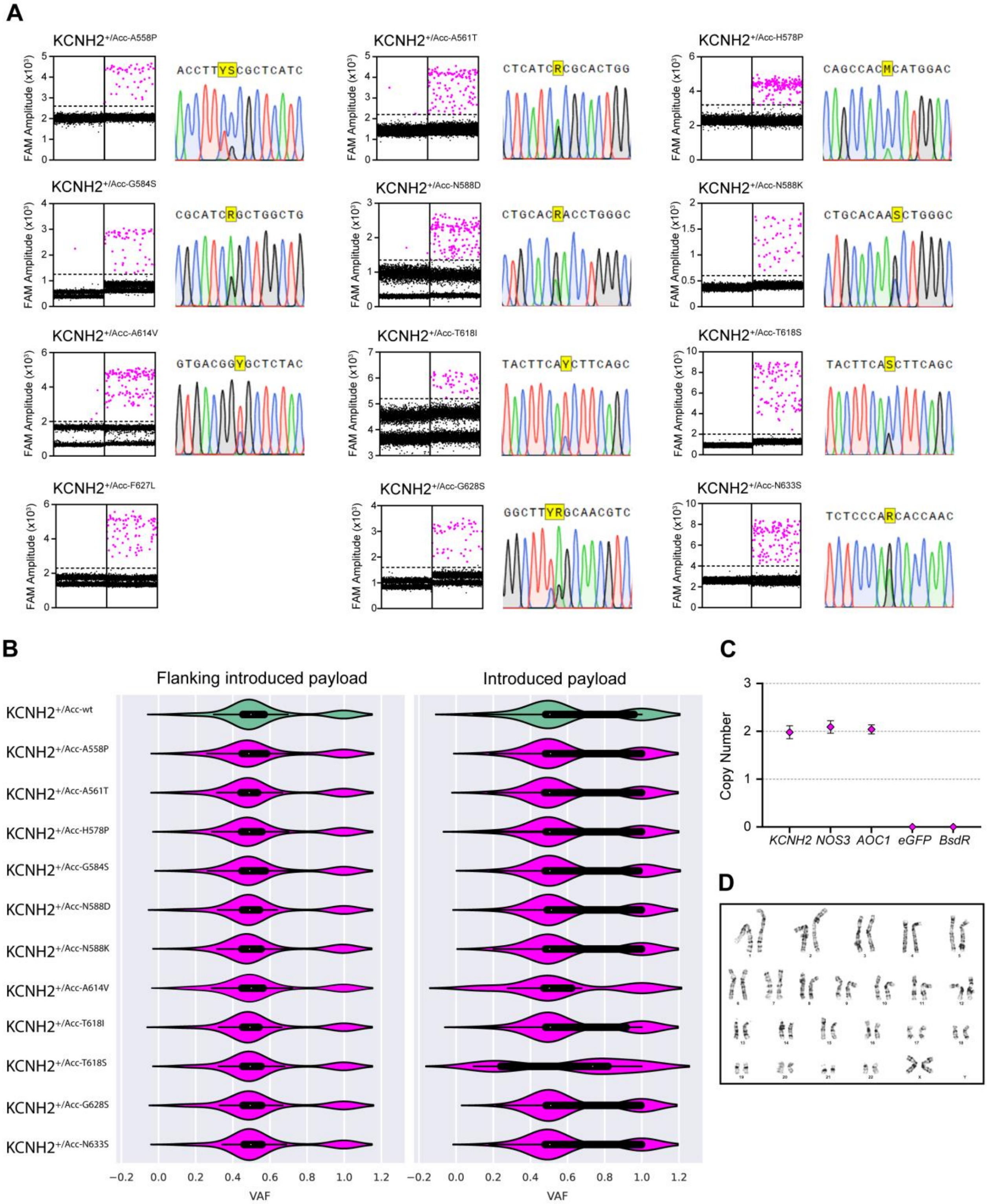

Figure S6. Characterising the *KCNH2*-variant hiPSCs. Related to Figure 6.

(A) Dot plots indicate the detection by ddPCR following Bxb1-mediated integration of the specified *KCNH2* variants (A558P; A561T; H578P; G584S; N588D; N588K; A614V; T618I; T618S; F627L; G628S; N633S) in a pool of transfected cells. Chromatograms show subsequent Sanger sequence analysis following Cre transfection and single cell deposition to subclone and demultiplex the variants. Note, while the variant

KCNH2$^{+/Acc\text{-}F627L}$ was detected as integrated in the pool of transfected cells, clonal lines were not recovered hence why no chromatogram is available.

(B) VAF analysis of *KCNH2*$^{+/Acc\text{-}wt}$ and the 11 *KCNH2*-variant hiPSC lines confirmed heterozygous variants were present at a frequency of ~0.5 in the genomic regions flanking *KCNH2 (left*), as well as for the re-introduced copy of *KCNH2 (right)* apart for the variant KCNH2-T618S for which a rearrangement appears to have occurred in the hiPSC line.

(C) ddPCR confirming that a *KCNH2*$^{+/Acc\text{-}A561T}$ hiPSC line generated by STRAIGHT-IN contained 2 copies of genomic genes *KCNH2*, *NOS3* and *AOC1*, and no copies of the Bxb1-LP cassette transgenes, *eGFP* and *BsdR.* Error bars represent Poisson 95% CI.

(D) G-banding karyogram for the *KCNH2*$^{+/Acc\text{-}A561T}$ hiPSC line indicating a normal 46, XX karyotype.

Table S1. KCNH2 variants introduced in KCNH2$^{+/Acc}$ hiPSCs, annotated and with corresponding gBlock sequence. Related to Figure 6 and STAR Methods.

SNP	Nucleotide change	Protein change	Interpretation (ClinVar)	Associated Disease	gBlock	Comments
rs121912576	c.1672G>C	A558P	pathogenic	Long QT syndrome	cttgccccccttgccccatcaacggaatgtgccccttccctgtcccccagctgatcgggctgctgaagactgcgcggctgctgcggctgg<br>tgcgcgtggcgcggaagctggatcgctactcagagtacggcgcggccgtgctgttcttgctcatgtgcaccttCCgctcatcgcgcac<br>tggctagcctgcatctggtacgccatcggcaacatggagcagccacacatggactcacgcatcggctggctgcacaacctgggcgac<br>cagataggcaaaccctacaacagcagcggcctgggcggcccctccatcaaggacaagtatgtgacggcgctctacttcaccttcagc<br>agcctcaccagtgtgggcttcggcaacgtctctcccaacaccaactcagagaagatcttctccatctgcgtcatgctcattggctgtgag<br>tgtgcccaggggcgggcggcggggagagcccacggtggaggaacccaagttggaggaaactgaggctgctagccgggcca	silent mutation introduced into adjacent amino acid
rs199472921	c.1681G>A	A561T	pathogenic	Long QT syndrome	cttgccccccttgccccatcaacggaatgtgccccttccctgtcccccagctgatcgggctgctgaagactgcgcggctgctgcggctgg<br>tgcgcgtggcgcggaagctggatcgctactcagagtacggcgcggccgtgctgttcttgctcatgtgcacctttgcgctcatcAcgcac<br>tggctagcctgcatctggtacgccatcggcaacatggagcagccacacatggactcacgcatcggctggctgcacaacctgggcgac<br>cagataggcaaaccctacaacagcagcggcctgggcggcccctccatcaaggacaagtatgtgacggcgctctacttcaccttcagc<br>agcctcaccagtgtgggcttcggcaacgtctctcccaacaccaactcagagaagatcttctccatctgcgtcatgctcattggctgtgag<br>tgtgcccaggggcgggcggcggggagagcccacggtggaggaacccaagttggaggaaactgaggctgctagccgggcca	
rs794728376	c.1733 A>C	H578P	uncertain significance	-	cttgccccccttgccccatcaacggaatgtgccccttccctgtcccccagctgatcgggctgctgaagactgcgcggctgctgcggctgg<br>tgcgcgtggcgcggaagctggatcgctactcagagtacggcgcggccgtgctgttcttgctcatgtgcacctttgcgctcatcgcgcact<br>ggctagcctgcatctggtacgccatcggcaacatggagcagccacCcatggactcacgcatcggctggctgcacaacctgggcgacc<br>agataggcaaaccctacaacagcagcggcctgggcggcccctccatcaaggacaagtatgtgacggcgctctacttcaccttcagca<br>gcctcaccagtgtgggcttcggcaacgtctctcccaacaccaactcagagaagatcttctccatctgcgtcatgctcattggctgtgagt<br>gtgcccaggggcgggcggcggggagagcccacggtggaggaacccaagttggaggaaactgaggctgctagccgggcca	
rs199473428	c.1750G>A	G584S	likely pathogenic; pathogenic	Long QT syndrome	cttgccccccttgccccatcaacggaatgtgccccttccctgtcccccagctgatcgggctgctgaagactgcgcggctgctgcggctgg<br>tgcgcgtggcgcggaagctggatcgctactcagagtacggcgcggccgtgctgttcttgctcatgtgcacctttgcgctcatcgcgcact<br>ggctagcctgcatctggtacgccatcggcaacatggagcagccacacatggactcacgcatcAgctggctgcacaacctgggcgacc<br>agataggcaaaccctacaacagcagcggcctgggcggcccctccatcaaggacaagtatgtgacggcgctctacttcaccttcagca<br>gcctcaccagtgtgggcttcggcaacgtctctcccaacaccaactcagagaagatcttctccatctgcgtcatgctcattggctgtgagt<br>gtgcccaggggcgggcggcggggagagcccacggtggaggaacccaagttggaggaaactgaggctgctagccgggcca	
rs199473431	c.1762A>G	N588D	not provided	Long QT syndrome	cttgccccccttgccccatcaacggaatgtgccccttccctgtcccccagctgatcgggctgctgaagactgcgcggctgctgcggctgg<br>tgcgcgtggcgcggaagctggatcgctactcagagtacggcgcggccgtgctgttcttgctcatgtgcacctttgcgctcatcgcgcact<br>ggctagcctgcatctggtacgccatcggcaacatggagcagccacacatggactcacgcatcggctggctgcacGacctgggcgacc<br>agataggcaaaccctacaacagcagcggcctgggcggcccctccatcaaggacaagtatgtgacggcgctctacttcaccttcagca<br>gcctcaccagtgtgggcttcggcaacgtctctcccaacaccaactcagagaagatcttctccatctgcgtcatgctcattggctgtgagt<br>gtgcccaggggcgggcggcggggagagcccacggtggaggaacccaagttggaggaaactgaggctgctagccgggcca	
rs104894021	c.1764C>G	N588K	pathogenic	Short QT syndrome	cttgccccccttgccccatcaacggaatgtgccccttccctgtcccccagctgatcgggctgctgaagactgcgcggctgctgcggctgg<br>tgcgcgtggcgcggaagctggatcgctactcagagtacggcgcggccgtgctgttcttgctcatgtgcacctttgcgctcatcgcgcact<br>ggctagcctgcatctggtacgccatcggcaacatggagcagccacacatggactcacgcatcggctggctgcacaaGctgggcgacc<br>agataggcaaaccctacaacagcagcggcctgggcggcccctccatcaaggacaagtatgtgacggcgctctacttcaccttcagca<br>gcctcaccagtgtgggcttcggcaacgtctctcccaacaccaactcagagaagatcttctccatctgcgtcatgctcattggctgtgagt<br>gtgcccaggggcgggcggcggggagagcccacggtggaggaacccaagttggaggaaactgaggctgctagccgggcca	

Table S1 *(continued)*

SNP	Nucleotide change	Protein change	Interpretation (ClinVar)	Associated Disease	gBlock	Comments
rs199472944	c.1841C>T	A614V	pathogenic	Long QT syndrome	cttgccccccttgccccatcaacggaatgtgccccttccctgtcccccagctgatcgggctgctgaagactgcgcggctgctgcggctgg<br>tgcgcgtggcgcggaagctggatcgctactcagagtacggcgcggccgtgctgttcttgctcatgtgcacctttgcgctcatcgcgcact<br>ggctagcctgcatctggtacgccatcggcaacatggagcagccacacatggactcacgcatcggctggctgcacaacctgggcgacc<br>agataggcaaaccctacaacagcagcggcctgggcggcccctccatcaaggacaagtatgtgacggTgctctacttcaccttcagca<br>gcctcaccagtgtgggcttcggcaacgtctctcccaacaccaactcagagaagatcttctccatctgcgtcatgctcattggctgtgagt<br>gtgcccaggggcgggcggcggggagagcccacggtggaggaacccaagttggaggaaactgaggctgctagccgggcca	
rs199472947	c.1853C>T	T618I	not provided	Short QT syndrome	cttgccccccttgccccatcaacggaatgtgccccttccctgtcccccagctgatcgggctgctgaagactgcgcggctgctgcggctgg<br>tgcgcgtggcgcggaagctggatcgctactcagagtacggcgcggccgtgctgttcttgctcatgtgcacctttgcgctcatcgcgcact<br>ggctagcctgcatctggtacgccatcggcaacatggagcagccacacatggactcacgcatcggctggctgcacaacctgggcgacc<br>agataggcaaaccctacaacagcagcggcctgggcggcccctccatcaaggacaagtatgtgacggcgctctacttcaTcttcagca<br>gcctcaccagtgtgggcttcggcaacgtctctcccaacaccaactcagagaagatcttctccatctgcgtcatgctcattggctgtgagt<br>gtgcccaggggcgggcggcggggagagcccacggtggaggaacccaagttggaggaaactgaggctgctagccgggcca	
rs199472947	c.1853C>G	T618S	not provided	Long QT syndrome	cttgccccccttgccccatcaacggaatgtgccccttccctgtcccccagctgatcgggctgctgaagactgcgcggctgctgcggctgg<br>tgcgcgtggcgcggaagctggatcgctactcagagtacggcgcggccgtgctgttcttgctcatgtgcacctttgcgctcatcgcgcact<br>ggctagcctgcatctggtacgccatcggcaacatggagcagccacacatggactcacgcatcggctggctgcacaacctgggcgacc<br>agataggcaaaccctacaacagcagcggcctgggcggcccctccatcaaggacaagtatgtgacggcgctctacttcaGcttcagca<br>gcctcaccagtgtgggcttcggcaacgtctctcccaacaccaactcagagaagatcttctccatctgcgtcatgctcattggctgtgagt<br>gtgcccaggggcgggcggcggggagagcccacggtggaggaacccaagttggaggaaactgaggctgctagccgggcca	
rs199473039	c.1881C>A/G	F627L	pathogenic	Long QT syndrome	cttgccccccttgccccatcaacggaatgtgccccttccctgtcccccagctgatcgggctgctgaagactgcgcggctgctgcggctgg<br>tgcgcgtggcgcggaagctggatcgctactcagagtacggcgcggccgtgctgttcttgctcatgtgcacctttgcgctcatcgcgcact<br>ggctagcctgcatctggtacgccatcggcaacatggagcagccacacatggactcacgcatcggctggctgcacaacctgggcgacc<br>agataggcaaaccctacaacagcagcggcctgggcggcccctccatcaaggacaagtatgtgacggcgctctacttcaccttcagca<br>gcctcaccagtgtgggcttGggcaacgtctctcccaacaccaactcagagaagatcttctccatctgcgtcatgctcattggctgtgag<br>tgtgcccaggggcgggcggcggggagagcccacggtggaggaacccaagttggaggaaactgaggctgctagccgggcca	
rs121912507	c.1882G>A	G628S	pathogenic	Long QT syndrome	cttgccccccttgccccatcaacggaatgtgccccttccctgtcccccagctgatcgggctgctgaagactgcgcggctgctgcggctgg<br>tgcgcgtggcgcggaagctggatcgctactcagagtacggcgcggccgtgctgttcttgctcatgtgcacctttgcgctcatcgcgcact<br>ggctagcctgcatctggtacgccatcggcaacatggagcagccacacatggactcacgcatcggctggctgcacaacctgggcgacc<br>agataggcaaaccctacaacagcagcggcctgggcggcccctccatcaaggacaagtatgtgacggcgctctacttcaccttcagca<br>gcctcaccagtgtgggcttTAgcaacgtctctcccaacaccaactcagagaagatcttctccatctgcgtcatgctcattggctgtgagt<br>gtgcccaggggcgggcggcggggagagcccacggtggaggaacccaagttggaggaaactgaggctgctagccgggcca	silent mutation introduced into adjacent amino acid
rs199472961	c.1898A>G	N633S	pathogenic	Long QT syndrome	cttgccccccttgccccatcaacggaatgtgccccttccctgtcccccagctgatcgggctgctgaagactgcgcggctgctgcggctgg<br>tgcgcgtggcgcggaagctggatcgctactcagagtacggcgcggccgtgctgttcttgctcatgtgcacctttgcgctcatcgcgcact<br>ggctagcctgcatctggtacgccatcggcaacatggagcagccacacatggactcacgcatcggctggctgcacaacctgggcgacc<br>agataggcaaaccctacaacagcagcggcctgggcggcccctccatcaaggacaagtatgtgacggcgctctacttcaccttcagca<br>gcctcaccagtgtgggcttcggcaacgtctctcccaGcaccaactcagagaagatcttctccatctgcgtcatgctcattggctgtgagt<br>gtgcccaggggcgggcggcggggagagcccacggtggaggaacccaagttggaggaaactgaggctgctagccgggcca	

Introduced mutations are indicated in capital letters.

Table S2. Sequences of oligonucleotides and gBlocks used for vector construction. Related to STAR Methods.

Name	Sequence (5'-3')	Vector
lox257_pENTR_HiFi_Fwd	ataacttcgtataggagactttatacgaagttaatggATCCCCTATAGTGAGTCG	pENTR-eGFP-attP(bxb)-*bsdR
pENTR_HiFi_Rev	GACTGGCCGTCGTTTTAC	pENTR-eGFP-attP(bxb)-*bsdR
pENTR_loxP_HiFi_Fwd	cgacgttgtaaaacgacggccagtcGCTAGCGAGCTCGGCGCG	pENTR-eGFP-attP(bxb)-*bsdR
PGK_GFP_HiFi_Rev	gctcaccatggtggcCTGCAGGTCGAAAGGCCCG	pENTR-eGFP-attP(bxb)-*bsdR
PGK_GFP_HiFi_Fwd	tttcgacctgcaggccaccATGGTGAGCAAGGGCGAG	pENTR-eGFP-attP(bxb)-*bsdR
bxb_GFP_HiFi_Rev	gacaaaccacgtatatctAGACATGATAAGATACATTGATGAGTTTGG	pENTR-eGFP-attP(bxb)-*bsdR
SV40_bxb_HiFi_Fwd	tcttatcatgtctagatatacgtggtttgtctggtcaaccaccgcggtctcagtggtgtacggtacaaacccatatGCCAAGCCTTTGTCTCAAG	pENTR-eGFP-attP(bxb)-*bsdR
lox257_bpA_HiFi_Rev	tgtaatacgactcactataggggatccattaacttcgtataaagtctcctatacgaagttatGCCTCAGAAGCCATAGAG	pENTR-eGFP-attP(bxb)-*bsdR
bleo_FRT-pENTR_HiFi_Fwd	gaagttcctattccgaagttcctattcttcaaatagtataggaacttcgaaCATCCAGCTGATATCCCC	pENTR-mCherry-attP(C31)-*bleoR
pENTR_HiFibuild_Rev	GACTGGCCGTCGTTTTAC	pENTR-mCherry-attP(C31)-*bleoR
pENTR_FRT_HiFibuild_Fwd	cgacgttgtaaaacgacggccagtcTTAATTAAGAAGTTCCTATTCCGAAGTTC	pENTR-mCherry-attP(C31)-*bleoR
PGK_cherry_HiFi_Rev	tcaccatggtggcaattGTCGAAAGGCCCGGAGAT	pENTR-mCherry-attP(C31)-*bleoR
PGK_cherry_HiFi_Fwd	cgggcctttcgacaattgccaccATGGTGAGCAAGGGCGAG	pENTR-mCherry-attP(C31)-*bleoR
C31_cherry_HiFi_Rev	gttggggcactacggatCCATAGAGCCCACCGCA	pENTR-mCherry-attP(C31)-*bleoR
C31_bleo_HiFibuild_Fwd	tgggctctatggatccgtagtgccccaactggggtaacctttgagttctctcagttgggggcgtaggcgcGCCAAGTTGACCAGTGCC	pENTR-mCherry-attP(C31)-*bleoR
pENTR_F3_HiFi_Rev	cactataggggatatcagctggatgttcgaagttcctatactatttgaagaataggaacttcggaataggaacttcATAAGATACATTGATGAGTTTGGACAAAC	pENTR-mCherry-attP(C31)-*bleoR
lox_eGFP_attP(Bxb)_lox257_Fwd	ctccaccccacagtggggcaAGTCACGACGTTGTAAAACGACG	AAVS1-Bxb1-LP-TC
lox_eGFP_attP(Bxb)_lox257_Rev	accaatcctgtccctagtaATGACCATGTAATACGACTCACTATAGGG	AAVS1-Bxb1-LP-TC
FRT_mCherry_attP(C31)_F3_Fwd	ctccaccccacagtggggcaAGTCACGACGTTGTAAAACGACG	AAVS1-φC31-LP-TC
FRT_mCherry_attP(C31)_F3_Rev	accaatcctgtccctagtaATGACCATGTAATACGACTCACTATAGGG	AAVS1-φC31-LP-TC
bxb_attB_lox gBlock	CCATTTCAGGTGTCGTGAGGAATTCGCCACCATGGCCGGCTTGTCGACGACGGCGGTCTCCGTCGTCAGGATCATCCGGATCCATAACTTCGTAT AGCATACATTATACGAAGTTATCATGATATTCGGCAAGCAGGCATCGACTAGTTAATTAAGCTAGCGCTGCAAGAACTCTTCCTCACGATAACTTC GTATAGGAGACTTTATACGAAGTTAAGCGCTCACTGGCCGTCGTTTTACA	pBR-bxb_attB donor
C31_attB_FRT gBlock	AATTCGCCACCATGGCCTCGAAGCCGCGGTGCGGGTGCCAGGGCGTGCCCTTGGGCTCCCCGGGCGCGTACTCCACCTCACCCATCGGATCCGA AGTTCCTATTCCGAAGTTCCTATTCTCTAGAAAGTATAGGAACTTCATGATATTCGGCAAGCAGGCATCGACTAGTTAATTAAGCTAGCGCTGCAA GAACTCTTCCTCACGGATCCGAAGTTCCTATTCCGAAGTTCCTATTCTTCAAATAGTATAGGAACTTCAGCGCTCACTGGCCGTCGTTTTACA	pBR-C31_attB donor
p15-SV40_HiFi_Fwd	atggttgctgactaattgagatgcaGCGCTAGCGGAGTGTATACTG	p15-bxb_attB donor
amp-lox257_HiFi_Rev	taatagcgaagaggcccgcaccgatACGTCAGGTGGCACTTTTCG	p15-bxb_attB donor
NdeI-BpiI-EF1a_Fwd	catatgaagacaatgccATTGGCTCCGGTGCCCGTC	pBR-attB(bxb)_ccdB_lox
NdeI-BpiI_neo4_Rev	catatgaagacaagctaCGATGCCTGCTTGCCGAATATCATG	pBR-attB(bxb)_ccdB_lox
XhoI-BpiI-Esp3I_lacZ_Fwd	ctcgagaagacaatagcagagACGACAGGTTTCCCGACTGGAAAGC	pBR-attB(bxb)_ccdB_lox
XhoI-BpiI-Esp3I-lacZ_Rev	ctcgagaagacaactccagagacGGTGTCGGGGCTGGCTTAAC	pBR-attB(bxb)_ccdB_lox
XhoI-BpiI_puro153_Fwd	ctcgagaagacaaggaGCTGCAAGAACTCTTCCTCACG	pBR-attB(bxb)_ccdB_lox
XhoI-BpiI_M13_Rev	ctcgagaagacaatcccTGTAAAACGACGGCCAGT	pBR-attB(bxb)_ccdB_lox
HpaI-Esp3I-BpiI-ccdB_Fwd	gttaacgtctcctagctgcctagtcttcACACATAACCAGGAGGTCAG	pBR-attB(bxb)_ccdB_lox
HpaI-Esp3I-BpiI-ccdB_Rev	gttaacgtctcactcctcccatgtcttcTGAAGTCAGCCCCATACGAT	pBR-attB(bxb)_ccdB_lox
BAC_EF1a_recom_Fwd	catccgatgcaagtgtgtcgctgtcgacggtgaccctatagtcgagggacCTTAATTCCCTCCCCAGC	BAC_attB(bxb)
BAC_SV40_recom_Rev	gctccgagaacgggtgcgcatagaaattgcatcaacgcatatagcgctagGATCCAGACATGATAAGATACATTG	BAC_attB(bxb)
KCNH2_2kb_recom-Fwd	aagatgctgatgactatgaataataaataattatcctgaggagaactccaGCTGCAAGAACTCTTCCTCACG	2-15 kb DNA payload donors
KCNH2_2kb_recom-Rev	gtagcagctgcaggacagtggccatgtctgcactcagccgggtctccagcCGATGCCTGCTTGCCGAATATCATG	2 kb DNA payload donor

Table S2 *(continued)*

Name	Sequence (5'-3')	Vector
KCNH2_5kb_recom-Rev	aggtgcaggcagatgtcagcctgcaggcactcagggaagcccttcagcacCGATGCCTGCTTGCCGAATATCATG	5 kb DNA payload donor
KCNH2_10kb_recom-Rev	ctgggccgcagagcccctgtcctgctcgccttcccggctggggccgccatCGATGCCTGCTTGCCGAATATCATG	10 kb DNA payload donor
KCNH2_15kb_recom-Rev	catcctcgttcttcacgggcaccacatccaccagacataggaagcagctccCGATGCCTGCTTGCCGAATATCATG	15 kb DNA payload donor
KCNH2_50kb_recom_Fwd	ctagcacctggaggtgtgtgaggggccaggatggactgcggatagaccagtCGCTGCAAGAACTCTTCCTCACG	p15-attB_KCNH2_wt_donor
KCNH2_50kb_recom_Rev	aagggggcagggcctgtcagatggatccctgacaaccatccgtctcaagtCGATGCCTGCTTGCCGAATATCATG	p15-attB_KCNH2_wt_donor
KCNH2_80b_M13_Fwd	aggtgccctgtcttgggctctgaaggccctacatacaaagtcatcctatctgctgaggcaaagatataaggttacctcgTGTAAAACGACGGCCAGTC	KCNH2-Bxb1-LP-TC
KCNH2_80bp_lox257_Rev	catttacatctgggccacagtgtccctccgtctccctctggccccggagcacatggccatctggttgtgctgactgtgctgCTATAGGGGATCCATTAACTTCG	KCNH2-Bxb1-LP-TC
KCNH2_ex7-amp_rec_Fwd	cttgccccccttgccccatcaacggaatgtgccccttccctgtcccccagCTAGCGCTTTGTTTATTTTTC	BAC with ccdB-amp cassette
KCNH2_ex7-ccdB_rec_Rev	tggcccggctagcagcctcagtttcctccaacttgggttcctccaccgtgAGCCCCATACGATATAAGTTG	BAC with ccdB-amp cassette
KCNH2_ex7_Fwd	CTTGCCCCCCTTGCCCCATC	p15-attB_KCNH2_variant_donors
KCNH2_ex7_Rev	TGGCCCGGCTAGCAGCCTC	p15-attB_KCNH2_variant_donors

Overhangs of oligonucleotides are indicated in lower case

Table S3. Sequences of oligonucleotides used for genotyping PCRs. Related to STAR Methods.

Purpose	Sequence Forward Primer (5' - 3')	Sequence Reverse Primer (5' - 3')
*AAVS1*_Bxb1 targeting – 5’ junction	CCGGAACTCTGCCCTCTAA	CCATGATATAGACGTTGTGGCTGTTG
*AAVS1*_Bxb1 targeting – 3’ junction	CGGCAGTTGGGATTCGTGAATTG	GTGAGTTTGCCAAGCAGTCA
*AAVS1*_φC31 targeting – 5’ junction	CCGGAACTCTGCCCTCTAA	CCGTCCTCGAAGTTCATCAC
*AAVS1*_φC31 targeting – 3’ junction	CCCACAACGAGGACTACACCATC	GTGAGTTTGCCAAGCAGTCA
Bxb1 Donor vector integration (attR) – *AAVS1*	CAAGATCCGCCACAACATCGAGGAC	CGATGCCTGCTTGCCGAATATCATG
Bxb1 Donor vector integration (attL) – *AAVS1*	TTTTGGAGTACGTCGTCTTTAGG	CTGGCAACTAGAAGGCACAGTCG
φC31 Donor vector integration (attR) – *AAVS1*	CATGGTGAGCAAGGGCGAGGAG	AGTCACGACGTTGTAAAACGAC
φC31 Donor vector integration (attL) – *AAVS1*	TTTTGGAGTACGTCGTCTTTAGG	GTGAAATTTGTGATGCTATTGC
BAC vector integration (attR) – *AAVS1*	GTGCTGCTGCCCGACAACCACTAC	GAACCTGCGTGCAATCCATCTT
BAC vector integration (attL) – *AAVS1*	TTTTGGAGTACGTCGTCTTTAGG	GTGAGTTTGCCAAGCAGTCA
Integration integrity – 2-15 kb payload	CTCGGCGCGCCATAACTTCG	GTGAGTTTGCCAAGCAGTCA
loxP cassette excision – *AAVS1*	CCGGAACTCTGCCCTCTAA	CGATGCCTGCTTGCCGAATATCATG
lox257 cassette excision – *AAVS1*	GCTGCAAGAACTCTTCCTCACG	GTGAGTTTGCCAAGCAGTCA
FRT cassette excision – *AAVS1*	CCGGAACTCTGCCCTCTAA	CGATGCCTGCTTGCCGAATATCATG
F3 cassette excision – *AAVS1*	GCTGCAAGAACTCTTCCTCACG	GTGAGTTTGCCAAGCAGTCA
Optogenetic loxP cassette excision – ASAP2f	TTCGGGTCACCTCTCACTCC	CTCACAGTCGTCTCCATGGT
Optogenetic lox257 cassette excision – ASAP2f	CAACATTCTGGGGCATAAACTGG	GGCTCCATCGTAAGCAAACC
Optogenetic loxP cassette excision – jRCaMP1b	TTCGGGTCACCTCTCACTCC	TTACGTCGCGATGAGTCGAC
Optogenetic lox257 cassette excision – jRCaMP1b	GGTCAGGTAAACTACGAAGAG	GGCTCCATCGTAAGCAAACC
Optogenetic loxP cassette excision – miRFP703	TTCGGGTCACCTCTCACTCC	TTCGCAATTCGAATGAGAGGC
Optogenetic lox257 cassette excision – miRFP703	GCTTCATCCGTTTCGAGCTG	GGCTCCATCGTAAGCAAACC
Optogenetic loxP cassette excision – AJMA	TTCGGGTCACCTCTCACTCC	CTCACAGTCGTCTCCATGGT
Optogenetic lox257 cassette excision – AJMA	CAACATTCTGGGGCATAAACTGG	GGCTCCATCGTAAGCAAACC
Integration integrity – ASAP2f-jRCaMP1b	CAACATTCTGGGGCATAAACTGG	TTACGTCGCGATGAGTCGAC
Integration integrity – jRCaMP1b-miRFP703	GGTCAGGTAAACTACGAAGAG	TTCGCAATTCGAATGAGAGGC
Integration integrity – miRFP703-ASAP2f	GCTTCATCCGTTTCGAGCTG	CTCACAGTCGTCTCCATGGT
KCNH2$^{+/Acc}$ targeting – 5’ junction	ACCCGGTCCTTGGTGTTTTC	CCATGATATAGACGTTGTGGCTGTTG
KCNH2$^{+/Acc}$ targeting – 3’ junction	CGGCAGTTGGGATTCGTGAATTG	ATTTCCAATCCTCAGGGGCG
Donor vector integration (attR) – *KCNH2*	ACCCGGTCCTTGGTGTTTTC	CGATGCCTGCTTGCCGAATATCATG
Donor vector integration (attL) – *KCNH2*	GCTGCAAGAACTCTTCCTCACG	ATTTCCAATCCTCAGGGGCG
loxP cassette excision – *KCNH2*	ACCCGGTCCTTGGTGTTTTC	CGATGCCTGCTTGCCGAATATCATG
lox257 cassette excision – *KCNH2*	GCTGCAAGAACTCTTCCTCACG	ATTTCCAATCCTCAGGGGCG
Screen KCNH2 exon 7 variants	CAAGGAGGCAGGTGGTGTAG	CCTCCAACTTGGGTTCCTCC

Table S4. Primer-Probe sets used for ddPCR. Related to STAR Methods.

Target Gene	Assay type	Primer/Probe	Sequence (5'- 3')	Fluorophore-Quencher	Source
eGFP	Copy number	Forward Primer<br>Reverse Primer<br>Probe	GCCGACAAGCAGAAGAACG<br>GGGTGTTCTGCTGGTAGTGG<br>AGATCCGCCACAACATCGAGG	FAM-ZEN-IBFQ	(Roberts et al., 2017)
BsdR	Copy number	Forward Primer<br>Reverse Primer<br>Probe	TGGCAACCTGACTTGTATCG<br>GTCCATCACTGTCCTTCACTATC<br>CGACAGGTGCTTCTCGATCTGCAT	FAM-ZEN-IBFQ	This study
Bxb1-*attP*	Integration	Forward Primer<br>Reverse Primer<br>Probe	GCATTCTAGTTGTGGTTTGTCC<br>ATGAGGGTGGATTCTTCTTGAG<br>CGTGGTTTGTCTGGTCAACCA	HEX-ZEN-IBFQ	This study
Bxb1-*attR*	Integration	Forward Primer<br>Reverse Primer<br>Probe	GCATTCTAGTTGTGGTTTGTCC<br>CTTAATTAACTAGTCGATGCCTGC<br>TCTCCGTCGTCAGGATCATCC	HEX-ZEN-IBFQ	This study
φC31-*attP*	Integration	Forward Primer<br>Reverse Primer<br>Probe	GAGGATTGGGAAGACAATAGC<br>AAGTCGTCCTCCACGAAG<br>CATGCTGGGGATGCGGTG	FAM-ZEN-IBFQ	This study
φC31-*attR*	Integration	Forward Primer<br>Reverse Primer<br>Probe	GAGGATTGGGAAGACAATAGC<br>CCTGCTTGCCGAATATCATG<br>ATCCGATGGGTGAGGTGGAG	FAM-ZEN-IBFQ	This study
KCNH2	Variant detection	Forward Primer<br>Reverse Primer<br>KCNH2_A561T Probe<br>KCNH2_A558P Probe<br>KCNH2_A614V Probe<br>KCNH2_H578P Probe<br>KCNH2_G584S Probe<br>KCNH2_N588D Probe<br>KCNH2_N588K Probe<br>KCNH2_T618I Probe<br>KCNH2_T618S Probe<br>KCNH2_F627L Probe<br>KCNH2_G628S Probe<br>KCNH2_N633S Probe	CTTGCCCCATCAACGG<br>GCACACTCACAGCCAATG<br>CGC TCA T+C+A C+GC ACT<br>ACCTT+C+CCGCTCA<br>TGA CG+G +T+GC TCT AC<br>AGC AGC C+AC +CC+A T<br>TCA CGC +AT+C +A+GC T<br>TGG CTG CAC +G+AC CT<br>CTG C+AC A+A+G +CTG G<br>ACT +T+CA +T+CT T+CA GC<br>CTT C+A+G +CTT +C+AG C<br>CT+T +GG+G C+AA CGT C<br>CTT +T+A+G CA+A CG+T CT<br>TCC C+A+G +CAC CA+A C	-<br>-<br>FAM-ZEN-IBFQ<br>FAM-ZEN-IBFQ<br>FAM-ZEN-IBFQ<br>FAM-ZEN-IBFQ<br>FAM-ZEN-IBFQ<br>FAM-ZEN-IBFQ<br>FAM-ZEN-IBFQ<br>FAM-ZEN-IBFQ<br>FAM-ZEN-IBFQ<br>FAM-ZEN-IBFQ<br>FAM-ZEN-IBFQ<br>FAM-ZEN-IBFQ	This study
KCNH2	Allele expression	Forward Primer<br>Reverse Primer<br>Wildtype allele probe<br>Introduced allele probe	CGTCCACTACTTCAAGGG<br>ACTCTGAGTAGCGATCCA<br>CAT CT+T +C+GG +CTC T<br>CAT CT+T +T+GG +C+TC T	-<br>-<br>FAM-ZEN-IBFQ<br>HEX-ZEN-IBFQ	This study

Table S4 *(continued)*

Target Gene	Assay type	Assay ID	Genomic Region	Fluorophore-Quencher	Source
RPP30	Copy number	dHsaCP2500350	hg19|chr10:92660373-92660495:+	HEX-IBFQ	Bio-Rad
KCNH2	Copy number	dHsaCNS652574025	hg19|chr7:150641909-150642031:+	FAM-IBFQ	Bio-Rad
NOS3	Copy number	dHsaCP1000146	hg19|chr7:150695400-150695522:+	FAM-IBFQ	Bio-Rad
AOC1	Copy number	dHsaCNS791538087	hg19|chr7:150549559-150549681:+	FAM-IBFQ	Bio-Rad
TMEM176B	Copy number	dHsaCNS938119842	hg19|chr7:150488415-150488537:+	FAM-IBFQ	Bio-Rad
ABCB8	Copy number	dHsaCNS429552585	hg19|chr7:150725518-150725640:+	FAM-IBFQ	Bio-Rad

+ symbol indicates the following nucleotide is a locked nucleic acid
